# Mental fatigue in golf: A systematic review

**DOI:** 10.1371/journal.pone.0310403

**Published:** 2025-02-20

**Authors:** Xiaoyang Pan, Kim Geok Soh, Wan Marzuki Wan Jaafar, Kim Lam Soh, Nuannuan Deng, Shudian Cao, Mingtian Li, Huange Liu

**Affiliations:** 1 Faculty of Educational Studies, Department of Sport Studies, Universiti Putra Malaysia, Selangor, Malaysia; 2 Faculty of Educational Studies, Department of Counsellor Education and Counselling Psychology, Universiti Putra Malaysia, Selangor, Malaysia; 3 Faculty of Medicine and Health Sciences, Department of Nursing, Universiti Putra Malaysia, Selangor, Malaysia; 4 School of Physical Education, Xihua University, Sichuan, China; Shahid Chamran University of Ahvaz, ISLAMIC REPUBLIC OF IRAN

## Abstract

Mental fatigue, or cognitive fatigue, is a multi-aspect of exhaustion resulting from prolonged engagement in mentally demanding tasks, characterized by diminished energy, mental exhaustion, and distraction, which can adversely impact various aspects of golfers’ performance. However, there are still limited systematic reviews on the interaction between mental fatigue and athletes’ performance in golf. This study aims to provide a comprehensive analysis of the correlation between mental fatigue and golf and demonstrate the current state of research and characterization of research in the field. The systematic review was conducted using a PRISMA flow chart, with thorough literature searches across PubMed, Web of Science, SPORTDiscus, Scopus, and China National Knowledge Infrastructure (CNKI) databases. The quality of the literature was assessed using Qualsyst. The study summarized findings from 10 articles on the interaction between mental fatigue and athletes’ performance in golf. It indicates that mental fatigue is influenced by factors such as duration of play and walking distance, with prolonged golf tasks inducing mental fatigue. Mental fatigue directly affects golf performance, including the overall score for 18 holes, iron club accuracy, drive distance, and especially impacts putting performance. However, the results of these studies are limited and one-sided because studies conducted on driving ranges or in laboratories focus only on putting performance and ignore other golf skills. The study on the effects of mental fatigue on iron accuracy and driving distance was conducted under competitive conditions on an outdoor golf course, making it impossible to eliminate confounding factors. The lack of intervention studies on other specific golf skills may limit a comprehensive understanding of the impact of mental fatigue on golf performance.

**Trial registration Systematic Review Registration:** [https://inplasy.com/][INPLASY202410111].

## Introduction

Mental fatigue (MF), or cognitive fatigue, arises from prolonged mental effort during demanding tasks [1]. It manifests as feeling mentally drained, lacking energy, exhaustion, and reduced motivation to focus [2–5]. MF is identified through neurophysiological, behavioral, or subjective indicators [6]. Athletes report symptoms impacting their physical, technical, tactical, psychological, and psychomotor performance [7,8]. Research indicates athletes see MF as detrimental to training and competition [9]. Athletes experience MF across all sports, particularly pre-season and in-season [10]. Recognizing MF’s impact on sports is crucial, and practitioners should manage symptoms properly [11].

Golf is a sport enjoyed globally by recreational and professional players [12]. Recently, there has been increasing scientific interest in how golfers manage MF [13]. Competitive golf requires exceptional hand-eye coordination, significant cognitive demands, advanced motor skills, biomechanical precision, and extended periods of play [14–18]. Research indicates that critical shot-making decisions, extensive walking, numerous high-intensity swings, and putting can induce MF, potentially impairing performance [19]. From the cognitive perspective, golf requires continuous effort, strategic thinking, planning, and adaptation to course conditions [20]. Prolonged engagement in these tasks may lead to cognitive fatigue, affecting decision-making [21]. The extended duration of golf rounds [22], involving continuous shot decisions and swings, exposes players to sustained mental strain, potentially leading to MF [23]. Complex decision-making in golf, such as choosing clubs, shot types, and putting techniques, can make golfers vulnerable to MF, impairing optimal decision-making and resulting in suboptimal performance [24]. The biomechanical complexity of swings and putts is susceptible to MF, influencing coordination and technical performance [25].

In summary, MF is important in training and competition [13]. The need for this systematic review lies in the fact that there is a lack of systematic reviews of the interactions between MF and golf performance. Although studies have been conducted to point out the potential effects of MF on golf sport performance, a systematic review can comprehensively summarize and analyze the existing findings to better understand the current state of research and findings in this area. By identifying gaps in existing research and research gaps, systematic reviews can provide guidance for future research and identify questions and directions that need to be further explored.

## Methods

This article adhered to the PRISMA (2020) guidelines [26]. This title has already been registered on the International Platform of Registered Systematic Review and Meta-analysis Protocols, and the registration number is INPLASY202410111. Electronic databases such as PubMed, Web of Science, SPORTDiscus, Scopes, and China National Knowledge Infrastructure (CNKI) were systematically searched until January 2, 2024. Boolean operators AND and OR were applied with the specified keywords, as shown in **Table 1** below. Harmonized search terms and search string are used in all databases (**S1 Table**).

**Table 1:** Search string.

Search Builder	Search String
**Mental Fatigue**	"mental fatigue" OR "cognitive fatigue" OR "mental effort" OR "cognitive effort" OR "mental exertion" OR "ego depletion"
**Golf**	golf

Population, intervention, comparison, outcome, and study design (PICOS) criteria were included (**Table 2**). Studies had to meet the following conditions: (1) Participants had to be healthy individuals engaged in golf; (2) Any type of intervention was considered; (3) Comparison group includes any different intervention or no intervention; (4) Outcomes included levels of MF or any form of golf performance, encompassing physical, technical, cognitive, and tactical aspects; and (5) The study designs needed to include randomized controlled trials (RCT), non-randomized controlled trials (nRCT), and non-randomized non-controlled trials (nCT).

**Table 2:** PICOS criteria for inclusion criteria.

Items	Detailed inclusion criteria
**Population**	Healthy people engaged in golf
**Intervention**	Any form of intervention
**Comparison**	Any different intervention or no intervention
**Outcome**	MF levels; or any form of golf performance
**Study designs**	RCT, nRCT and nCT

**Fig 1:**
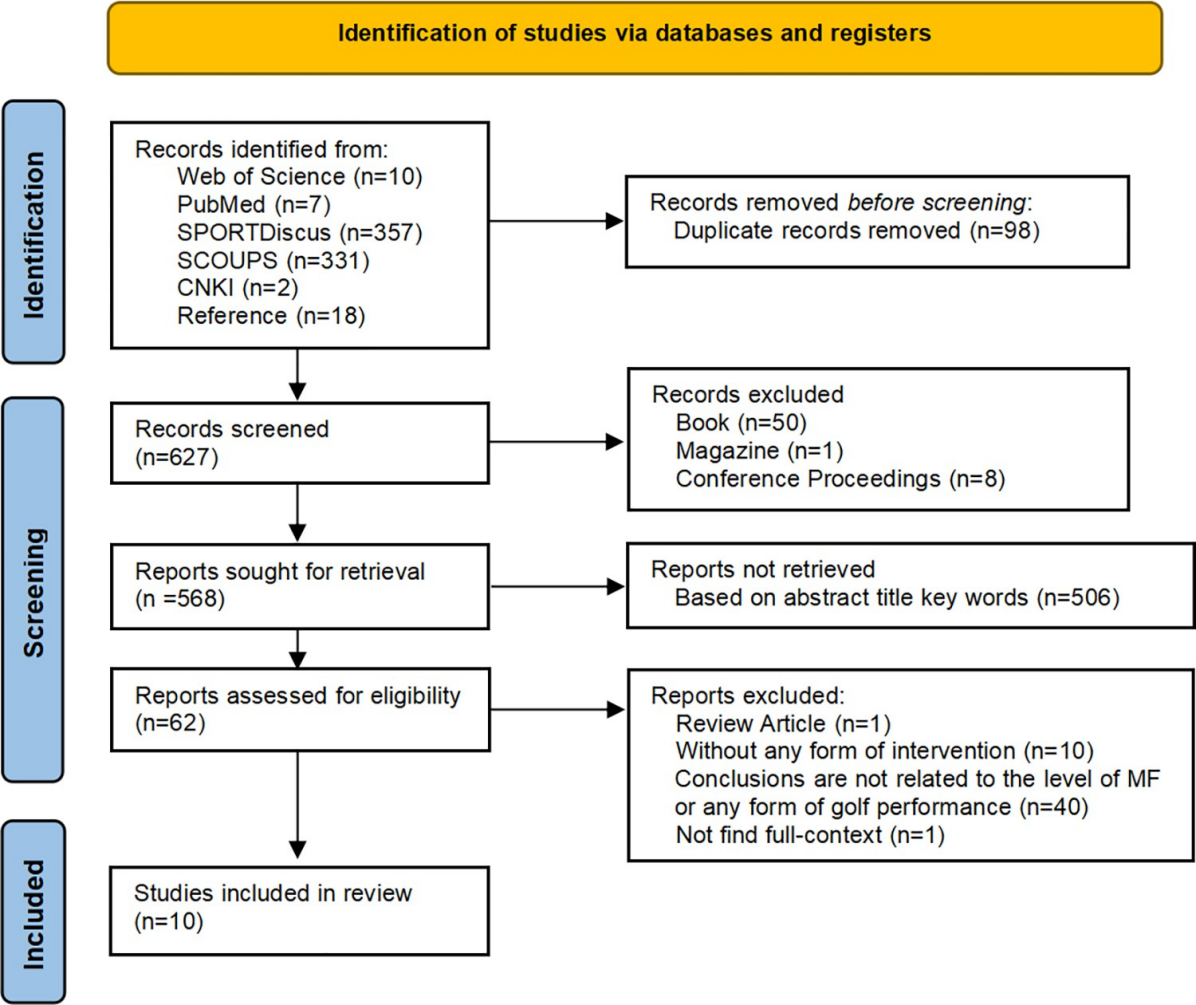
PRISMA flow diagram.

The results (titles and abstracts) of studies obtained using the search technique and those obtained from other sources to identify articles that may satisfy the inclusion criteria mentioned above were independently screened. The identification, screening, and inclusion phases of selecting references for analysis are summed up in **Fig 1**. Automation tools are not used in the process. Additionally, this review strictly adhered to the PRISMA Checklist requirements to ensure transparency and systematic procedures during the screening process (**S1 File**).

The methodology quality was assessed using "QualSyst" [27], which includes 14 items (**Table 3**). Each item was scored based on the degree to which specific criteria were met: yes = 2, partial = 1, no = 0. Items that did not apply to the study design were marked as "NA" and excluded from the total score calculation. A score of ≥75% indicated strong quality, 55–75% indicated moderate quality, and a ≤55% indicated weak quality. The detailed ratings are shown in **Table 3**.

**Table 3:** Assessing the quality of studies.

Criteria	Doan et al.,[28] 2007	Stevenson et al.,[19] 2009	Lam et al.,[29] 2010	Mumford et al.,[12] 2015	Shin et al.,[30] 2019	Campbell et al.,[31] 2019	Carnegie et al.,[32] 2020	Runswick et al.,[33] 2021	Galanis et al.,[34] 2022	Nagashima et al.,[35] 2023
**Ⅰ**	2	2	2	2	2	2	2	2	2	2
**Ⅱ**	2	2	2	2	2	2	2	2	2	2
**Ⅲ**	2	2	2	2	2	2	2	2	2	2
**Ⅳ**	2	2	2	2	2	2	2	2	2	1
**Ⅴ**	0	2	0	2	0	2	0	0	2	2
**Ⅵ**	0	0	0	0	0	0	0	0	0	0
**Ⅶ**	0	2	0	2	0	2	0	0	2	2
**Ⅷ**	2	2	2	2	2	2	2	2	2	2
**Ⅸ**	2	2	2	2	2	2	2	2	2	2
**Ⅹ**	2	2	2	2	2	2	2	2	2	2
**XI**	2	2	2	2	2	2	2	2	2	2
**XII**	0	0	0	0	0	1	0	0	1	0
**XIII**	2	2	2	2	2	2	2	2	2	2
**XIV**	2	2	2	2	2	2	2	2	2	2
**Rating**	Moderate	Strong	Moderate	Strong	Moderate	Strong	Moderate	Moderate	Strong	Strong

I, description of the problem; II, appropriate study design; III, appropriate subject selection; IV, characterization; V; V, random assignment; VI, VII, blinding of subjects; VIII, outcome measures are clear, and sample sizes are appropriate; X, analytic methods are well described; XI; XII, control for confounding; XIII, detailed report; XIV, conclusions supported by results. NA, not applicable; 2, for yes; 1, for partial, for no.

## Results

**Fig 1** Flowchart depicting the study selection process from search to inclusion in the review. Studies that met the inclusion criteria but were excluded are labeled in the flow. 725 articles were screened in the review and 10 articles were finally included (**S2 Table**). The quality evaluation results showed that five articles were of strong quality, five were of moderate quality, and none were of weak quality (**Table 3**). **Table 4** analyses the characteristics of these ten articles. Based on the country or region of the author’s university, five articles were from Europe (three from the United Kingdom, one each from Greece and Ireland)[19,31–34], two articles were from North America (both from the United States) [12,28], and three articles were from Asia (one each from Japan, South Korea, and Hong Kong, China)[29,30,35].

**Table 4:** Characteristics of the 10 articles.

**Author** **(Year)** **Country**	**Gender**	**Age**	**Handicap**	**Mental fatigue intervention modalities**	**Duration**	**Measurement**	**Evaluating the performance**	**Study designs**	**Results of the researches**	
									**Sports psychology**	**Golf performance**
Brandon Doan (2007) [28] United States	8 M	20.3 [± 1.5] years	4.4 ± 1.2	36 consecutive holes of official competition	~600 minutes	Visual Analogue Scales	36-hole tournament	nCT	MF ↑ in I vs. C. PF ↑ in I vs. C. CSAI-2 somatic anxiety ↑ in I vs. C.	Total scores ↓ in I vs. C.
Emma J. Stevenson (2009) [19] United Kingdom	20 M	23 [± 4] years	15.0 ± 4.0	Carbohydrate-electrolyte formulation containing caffeine	~240 minutes	Visual Analogue Scales	Putt during a round of simulated golf	RCT, Crossover	Alertness and relaxation ↑ in I vs. C. MF ↓ in I vs. C.	2 m putting performance ↑ in I vs. C. 5 m putting performance ↑ in I vs. C.
Wing Kai Lam (2010) [29] Hong Kong	11 M 16 F	21.3 [± 2.95] years	Beginner	Putt following a successful putt (Preparation, Execution)	~30 minutes	Cognitive Performance	1.5-m putt	nCT	Cognitive effort ↓ in I vs. C. Movement times ↓ in I vs. C.	1.5 m putting performance ↑ in I vs. C.
Petey W. Mumford (2015) [12] United States	12 M	34.8 [± 13.9] years	6.5 ± 3.5	Caffeine-containing supplement	~240 minutes	Cognitive Performance	36-hole tournament	RCT, Crossover	Alertness, Overall confidence, Concentration → in I vs. C. MF ↓ in I vs. C.	FIR, putts/round, shots hit OB, sand shots, SS%, first putt distance → in I vs. C. Iron club accuracy, drive distance ↑ in I vs. C. Total score ↑ in I vs. C.
Myoungjin Shin (2019) [30] South Korea	51 F	22.25 [± 1.78] years	Beginner	3 minutes of neutral transcription followed by 7 minutes of transcription with the omission of "e" and "t"	10 minutes	Questionnaire	4-m and 6-m putt	RCT	Ego depletion or anxiety ↑ in I vs. C.	Putting performance ↓ in I vs. C.
Mark J. Campbell (2019) [31] Ireland	24 M	38.72 [± 11.62] years	H 6.9 ± 2.3 and L 17.0 ± 3.6	Low handicap	~30 minutes	Cognitive Performance	1.83-m and 3.66-m putt	nRCT	Cognitive effort → in I vs. C.	Putting performance → in I vs. C.
Evelyn Carnegie (2020) [32] United Kingdom	11 M 3 F	M 26.3 [± 9.9] years and F 27.4 [± 10.1] years	H 5.6 ± 2.6 and L 17.3 ± 3.9	Low handicap	~10 minutes	Cognitive Performance	1.75-m right to left, left to right and straight putt	nRCT	Not	Putting performance ↑ in I vs. C. Visual search behavior ↓ in I vs. C. Percentage viewing time ↓ in I vs. C. QE duration → in I vs. C.
**Authors (Year) Country**	**Gender**	**Age**	**Handicap**	**Mental fatigue intervention modalities**	**Duration**	**Measurement**	**Sport-skill Task**	**Study designs**	**Sports psychology**	**Golf performance**
Oliver R. Runswick (2021) [33] United Kingdom	21	21.22 [± 1.89] years	Beginner	"If miss this putt, will lose the hole" pressure competition scenario	~10 minutes	Cognitive Performance	2.43-m putt	nCT	Not	Putting accuracy ↓ in I vs. C. Quiet Eye Duration ↑ in I vs. C. Pupillometry → in I vs. C.
Evangelos Galanis (2022) Exp1[34] Greece	32 M 30 F	18.58 [± 1.03] years	Beginner	Perform the golf-putting using self-talk before each putt	15 minutes	Questionnaire	2.5-m, 3-m and 2-m putt	RCT	Ego depletion ↓ in I vs. C. Attention ↑ in I vs. C.	Putting performance ↑ in I vs. C.
Evangelos Galanis (2022) Exp2[34] Greece	27 M 27 F	19.91 [±1.04] years	Beginner	Perform the golf-putting using self-talk before each putt	15 minutes	Questionnaire	2.5m, 3m and 2m putt when the flag is not waved	RCT	Ego depletion ↓ in I vs. C. Attention ↑ in I vs. C.	Putting performance ↑ in I vs. C.
Yosuke Nagashima (2023) [35] Japan	11	20.1 [± 1.0] years	Not	Carbohydrate supplemented gummies continuously ingested (CHO intake)	~240 minutes	Visual Analogue Scales	18-hole competition	RCT, Crossover	Self-perceived levels of fatigue ↓ in I vs. C. Self-perceived levels of concentration ↑ in I vs. C. Self-perceived levels of relaxation ↑ in I vs. C.	Scores, 2.5 m putting test, Club head speed, Driving distance, and Accuracy → in I vs. C.

M, male; F, female; H, high-handicap; L, low-handicap; I, intervention; C, control; RCT, randomized controlled trial; MF, mental fatigue

↑,Increase; ↓, Decrease; →, Unchanged; ~, Approximately.

### Participant characteristics

Out of ten articles, four studies focused on male golfers [12,19,28,31], one study focused on a female golfer [30], and three studies investigated mixed-gender populations [19,32,34]. Additionally, two studies did not specify the gender of the golfers [33,35]. Furthermore, MF effects varied across age groups: five studies involved participants aged 18–22 [28,29,33–35], three studies included golfers aged 22–30 [19,30,32], and two studies focused on golfers aged 30–50 [12,31]. The literature includes four studies involving beginner golfers [29,30,33,34], three studies focused on sub-professional golfers [12,19,31], and three studies on well-trained golfers [28,32,35].

### MF-inducing interventions

This systematic review included ten studies covering various MF interventions, including measures to induce MF and measures to recover from MF. As measures to induce MF, Shin et al. [30] required participants to transcribe a neutral text at their fastest speed participants in the experimental group had to omit all letters "e" and "t", inducing MF by violating habitual writing practices. Doan et al. [28] used a continuous 36-hole official competition and Runswick et al. [33] used a pressure competition scenario, simulating actual competition pressure to induce MF. Measures to recover from MF mainly included nutritional supplements and psychological strategies, such as Stevenson et al. [19] and Mumford et al.’s [12] caffeine-containing drinks and supplements, as well as Nagashima et al.’s [35] carbohydrate supplement gummies, aimed to alleviate MF, increase alertness, and reduce fatigue to improve performance. Psychological strategies aimed to improve performance by adjusting the athletes’ psychological state, such as Lam et al. [29] preparation and execution following a successful putt and Galanis et al. [34] self-talk strategy.

The MF intervention scenarios included a laboratory scenario, a simulated match scenario, and an actual match scenario. Three studies focused on match scenarios [12,28,35], one study investigated simulated match scenarios [19], and six studies investigated laboratory scenarios that focused on technique/skill, specifically putting performance [29,30,31,32,33,34] The short-duration studies are all from the laboratory, while the long durations are all based on competition scenarios. For example, six studies examined activities lasting 1–30 minutes [29,30,31,32,33,34], three studies investigated activities lasting 120–240 minutes [12,19,35] and one study explored activities lasting over 240 minutes [28].

Because of the different research scenarios, the tools for detecting MF are categorized in two ways. Five out of ten studies detected MF through subjective measures of Visual Analogue Scales [19,28,35] and Questionnaire [30,34]. The other five studies identified the effects of MF through Cognitive Performance [12,29,31,32,33].

### Effects of MF on golf performance

Doan et al. [28] demonstrated in their study that physical fatigue and MF significantly increased as the number of holes played in a 36-hole golf tournament progressed, leading to a decline in overall golf scores. Shin et al. [30] supported this observation in a laboratory setting, showing that MF significantly impacts putting performance. Campbell et al. [31] and Carnegie et al. [32] explored the potential moderating effects of different skill levels of golfers on intervention outcomes. While their study designs varied, their results indicated potential differences between high and low-level golfers in response to MF and performance.

Galanis et al. [34] found that self-talk strategies significantly reduced the occurrence of MF and markedly improved golf putting performance. This suggests that cognitive interventions can enhance athletes’ focus and performance levels.

On the other hand, Mumford et al. [12] highlighted the benefits of caffeine supplementation in enhancing alertness, confidence, and attention during golf competition, while reducing the experience of MF. Nagashima et al. [35] further supported this finding, showing that continuous ingestion of carbohydrate supplements lowered perceived fatigue levels and increased attention and relaxation. Stevenson et al. [19] confirmed that using carbohydrate-electrolyte formulations containing caffeine significantly improved golf putting accuracy.

## Discussion

### Participant characteristics

Only two studies focused on high-level athletes [12,28], and even four studies had participants who were beginners who had never been exposed to golf [29,30,33,34]. Because high-level athletes and beginners may exhibit significant differences in their responses to MF, intervention effects, and golf performance, results from studies on beginners cannot be directly generalized to high-level athletes. This limitation may restrict the generalizability of study findings. Focusing excessively on beginners or individuals who have never been exposed to golf may lead to gaps and shortcomings in theoretical development, intervention design, and practical application. To comprehensively understand the role of MF in golf, future research should prioritize investigating high-level athletes and conducting comparative analyses across different levels of athletes.

### MF-inducing interventions

Prolonged engagement in competitive golf, with its unique demands, may induce hormonal changes that impact immune function and influence overall performance on the course. Doan et al. [28] shed light on the potential consequences of extended golfing on hormonal balance, particularly the delicate interplay between testosterone and cortisol levels. Elevated testosterone levels, often associated with aggressive moods, might not align with the requirements for optimal golf performance. Interestingly, the study by Filaire et al. [36] found essential connections among cortisol levels, physical state anxiety, and cognitive state anxiety. Considering the complex relationship between MF and hormonal fluctuations [28], understanding these physiological mechanisms remains a research gap, which will be a feasible direction for future research, such as how changes in hormone levels affect specific golf performance and how interventions can optimize these effects.

According to Doan et al. [28], during a continuous 36-hole golf competition, golfers experienced an increase in MF with each successive hole, with significant increases in MF observed between holes 25–30 and 31–36. A possible reason for this is that a golf competition lasting over 5 hours requires athletes to engage in complex strategic thinking and decision-making. For instance, selecting the right club, deciding on shot strategies, and adjusting swing angles all demand high levels of cognitive load. Prolonged high cognitive load can lead to MF [37]. Furthermore, unlike sports such as basketball, soccer, and volleyball, which are played on standardized fields, golf courses are designed in vast natural environments [16]. Golfers must operate like snipers, facing challenges from nature and constantly adjusting their play based on factors such as the slope of the fairway, grass length, temperature, humidity, wind speed, and direction. This interesting phenomenon suggests that the competitive golf task in an outdoor setting can itself be a trigger for MF.

### Effects of MF on golf performance

This review demonstrates that MF directly affects golf performance, including the overall score for 18 holes, iron club accuracy, drive distance, and especially impacts putting performance. Among the ten articles included in this review, seven studies only involved testing of golf putting [19,29–34]. As a result, the findings are limited and one-sided. The other three studies focused on 18-hole scores [12,28,25], with two of these studies examining driving performance [12,35], and only one study involving iron club tests [35]. These studies were conducted during outdoor 18-hole competitions, making it impossible to rule out various confounding factors of the outdoor environment. For example, Doan et al. [28] found that both physical fatigue and MF were significantly affected by the number of holes played and the duration of time, indicating that as a golf competition progresses, both physical fatigue and MF gradually increase, leading to a decline in golf performance. However, due to the difficulty in clearly distinguishing between physical fatigue and MF in this context, it is challenging to demonstrate that MF is the direct cause of the decline in golf performance. Based on the study designs included in this review, an effective method to avoid confounding factors is to induce MF in a controlled practice range or laboratory setting using specific tasks or tests and then accurately assess golf performance through golf-specific tests. Unfortunately, the results of this review show that the only golf-specific skill tested in such a controlled environment is putting, which is only 1 of 14 clubs in a professional golfer’s bag. This represents a significant research gap, and future studies must test more clubs and techniques.

On the other hand, various studies suggest that the beneficial effects of caffeine can enhance cognitive function in tasks with high mental and physical demands, indicating that its effects are not limited to physical performance enhancement [12,38]. Stevenson et al. [19] found that dietary interventions, such as consuming sports drinks containing carbohydrates and caffeine, can positively impact mental function levels during golf activities, especially in putting. Ataka et al. [39] supported Stevenson et al.’s [19] findings, pointing out that caffeine intake can improve work performance by stimulating the central nervous system without increasing fatigue. For example, Judelson et al. [40] suggested that 100-mg caffeine significantly decreased lethargy/fatigue and increased vigor. Lorenzo Calvo et al. [41] indicated that coffee might improve attention. This highlights the potential impact of nutrition on mental function during golf competitions, warranting further exploration of nutritional interventions in the future.

## Conclusions

In conclusion, investigating MF within the context of golf based on findings from ten different studies offers valuable insights into its diverse effects on golf performance. The intensity of MF experienced during golf activities appears to be influenced by factors such as extended game duration, extensive walking distances, and environmental conditions. Hormonal dynamics, particularly the intricate interaction between testosterone and cortisol levels, contribute significantly to the nuanced relationship between MF and golf performance. Interventions such as dietary adjustments, caffeine supplementation, and stress management techniques are identified as potential strategies to modulate MF levels during a golf round. Regarding the impact of MF on golf performance, sustained participation in competitive golf can induce physical and MF, impacting both energy reserves and cognitive sharpness. Caffeine supplementation is viable to alleviate perceived fatigue, sustain energy levels, and enhance overall golf performance. While initial instances of MF, as indicated by ego depletion and anxiety, may initially impede performance, repeated exposure and recovery of self-control resources can mitigate these effects over time. Skilled golfers often exhibit lower cognitive load during golf tasks, resulting in more efficient and consistent performance outcomes.

## Limitations

Only published articles were included in this review. Therefore, the results of the study may be affected by publication bias. In addition, this study excluded qualitative research, which may have limited the understanding of golfers’ perceptions and experiences of MF. Three of the ten articles selected were conducted during an 18-hole tournament, an outdoor bustling environment which may have interfered with the findings. Finally, the lack of articles on golf-specific skill tests other than putting limits the overall understanding of the knowledge of the effects of MF on golf performance.

## Supporting information

S1 Table Detailed search strategy. (PDF)

S2 Table Numbered table of all studies. (PDF)

S1 File PRISMA checklist. (DOCX)

## References

[1] Lorist MM, Boksem MA, Ridderinkhof KR. Impaired cognitive control and reduced cingulate activity during mental fatigue. Cognitive Brain Research. 2005 Jul 1;24(2):199–205. doi: 10.1016/j.cogbrainres.2005.01.018 15993758

[2] Giulio CD, Daniele F, Tipton CM. Angelo Mosso and muscular fatigue: 116 years after the first Congress of Physiologists: IUPS commemoration. Advances in physiology education. 2006 Jun;30(2):51–7. doi: 10.1152/advan.00041.2005 16709733

[3] Boksem MA, Tops M. Mental fatigue: costs and benefits. Brain research reviews. 2008 Nov 1;59(1):125–39. doi: 10.1016/j.brainresrev.2008.07.001 18652844

[4] Chaudhuri A, Behan PO. Fatigue in neurological disorders. The lancet. 2004 Mar 20;363(9413):978–88. doi: 10.1016/S0140-6736(04)15794-2 15043967

[5] Qi P, Ru H, Gao L, Zhang X, Zhou T, Tian Y, et al. Neural mechanisms of mental fatigue revisited: New insights from the brain connectome. Engineering. 2019 Apr 1;5(2):276–86. 10.1016/j.eng.2018.11.025.

[6] Proost M, Habay J, De Wachter J, De Pauw K, Rattray B, Meeusen R, et al. How to tackle mental fatigue: a systematic review of potential countermeasures and their underlying mechanisms. Sports Medicine. 2022 Sep;52(9):2129–58. doi: 10.1007/s40279-022-01678-z 35543922

[7] Cao S, Geok SK, Roslan S, Sun H, Lam SK, Qian S. Mental fatigue and basketball performance: a systematic review. Frontiers in Psychology. 2022 Jan 10;12:819081. doi: 10.3389/fpsyg.2021.819081 35082736 PMC8784842

[8] Van Cutsem J, Marcora S, De Pauw K, Bailey S, Meeusen R, Roelands B. The effects of mental fatigue on physical performance: a systematic review. Sports medicine. 2017 Aug;47:1569–88. doi: 10.1007/s40279-016-0672-0 28044281

[9] Russell S, Jenkins D, Rynne S, Halson SL, Kelly V. What is mental fatigue in elite sport? Perceptions from athletes and staff. European journal of sport science. 2019 Nov 26;19(10):1367–76. doi: 10.1080/17461391.2019.1618397 31081474

[10] Abbott W, Brownlee TE, Naughton RJ, Clifford T, Page R, Harper LD. Changes in perceptions of mental fatigue during a season in professional under-23 English Premier League soccer players. Research in Sports Medicine. 2020 Oct 1;28(4):529–39. doi: 10.1080/15438627.2020.1784176 32602742

[11] Russell S, Jenkins DG, Halson SL, Kelly VG. Mental fatigue increases across a 16-week pre-season in elite female athletes. Journal of Science and Medicine in Sport. 2022 Apr 1;25(4):356–61. doi: 10.1016/j.jsams.2021.12.002 35027320

[12] Mumford PW, Tribby AC, Poole CN, Dalbo VJ, Scanlan AT, Moon JR, et al. Effect of caffeine on golf performance and fatigue during a competitive tournament. Medicine & Science in Sports & Exercise. 2016 Jan 1;48(1):132–8. doi: 10.1249/MSS.0000000000000753 26285020

[13] Russell S, Johnston RD, Stanimirovic R, Halson SL. Global practitioner assessment and management of mental fatigue and mental recovery in high‐performance sport: A need for evidence‐based best‐practice guidelines. Scandinavian Journal of Medicine & Science in Sports. 2024 Jan;34(1):e14491. 10.1111/sms.14491. 37728880

[14] Smith MF. The role of physiology in the development of golf performance. Sports medicine. 2010 Aug;40:635–55. doi: 10.2165/11532920-000000000-00000 20632736

[15] Smith MF, Newell AJ, Baker MR. Effect of acute mild dehydration on cognitive-motor performance in golf. The Journal of Strength & Conditioning Research. 2012 Nov 1;26(11):3075–80. doi: 10.1519/JSC.0b013e318245bea7 22190159

[16] Farrally MR, Cochran AJ, Crews DJ, Hurdzan MJ, Price RJ, Snow JT, et al. Golf science research at the beginning of the twenty-first century. Journal of sports sciences. 2003 Sep 1;21(9):753–65. doi: 10.1080/0264041031000102123 14579870

[17] Wang KP, Cheng MY, Chen TT, Huang CJ, Schack T, Hung TM. Elite golfers are characterized by psychomotor refinement in cognitive-motor processes. Psychology of Sport and Exercise. 2020 Sep 1;50:101739. 10.1016/j.psychsport.2020.101739.

[18] Broughton TS. Golf Brain: A Neuropsychological Study of Performance in Competition (Doctoral dissertation, George Fox University).

[19] Stevenson EJ, Hayes PR, Allison SJ. The effect of a carbohydrate–caffeine sports drink on simulated golf performance. Applied Physiology, Nutrition, and Metabolism. 2009 Aug;34(4):681–8. 10.1139/H09-057. 19767804

[20] Roberts LJ, Jackson MS, Grundy IH. The effects of cognitive interference during the preparation and execution of the golf swing. International Journal of Sport and Exercise Psychology. 2021 May 4;19(3):413–28. 10.1080/1612197X.2019.1674901.

[21] Thompson CJ, Fransen J, Skorski S, Smith MR, Meyer T, Barrett S, et al. Mental fatigue in football: is it time to shift the goalposts? An evaluation of the current methodology. Sports Medicine. 2019 Feb 14;49(2):177–83. doi: 10.1007/s40279-018-1016-z 30387071

[22] Berlin N, Cooke MB, Belski R. Nutritional considerations for elite golf: A narrative review. Nutrients. 2023 Sep 23;15(19):4116. doi: 10.3390/nu15194116 37836399 PMC10574085

[23] Vine SJ, Moore LJ, Wilson MR. Quiet eye training facilitates competitive putting performance in elite golfers. Frontiers in psychology. 2011 Jan 28;2:8. doi: 10.3389/fpsyg.2011.00008 21713182 PMC3111367

[24] Swann C, Crust L, Keegan R, Piggott D, Hemmings B. An inductive exploration into the flow experiences of European Tour golfers. Qualitative Research in Sport, Exercise and Health. 2015 Mar 15;7(2):210–34. 10.1080/2159676X.2014.926969.

[25] Konings MJ, Hettinga FJ. Pacing decision making in sport and the effects of interpersonal competition: a critical review. Sports Medicine. 2018 Aug;48:1829–43. doi: 10.1007/s40279-018-0937-x 29799094

[26] Page M. J., McKenzie J. E., Bossuyt P. M., Boutron I., Hoffmann T. C., Mulrow C. D., et al. (2021). Updating guidance for reporting systematic reviews: development of the PRISMA 2020 statement. Journal of clinical epidemiology, 134, 103–112. doi: 10.1016/j.jclinepi.2021.02.003 33577987

[27] Kmet LM, Cook LS, Lee RC. Standard quality assessment criteria for evaluating primary research papers from a variety of fields. 10.7939/R37M04F16.

[28] Doan BK, Newton RU, Kraemer WJ, Kwon YH, Scheet TP. Salivary cortisol, testosterone, and T/C ratio responses during a 36-hole golf competition. International journal of sports medicine. 2007 Jun;28(06):470–9. doi: 10.1055/s-2006-924557 17111317

[29] Lam WK, Masters RS, Maxwell JP. Cognitive demands of error processing associated with preparation and execution of a motor skill. Consciousness and cognition. 2010 Dec 1;19(4):1058–61. doi: 10.1016/j.concog.2008.11.005 21074112

[30] Shin M, Kim Y, Park S. Effects of state anxiety and ego depletion on performance change in golf putting: A hierarchical linear model application. Perceptual and Motor Skills. 2019 Oct;126(5):904–21. doi: 10.1177/0031512519856970 31219404

[31] Campbell MJ, Moran AP, Bargary N, Surmon S, Bressan L, Kenny IC. Pupillometry during golf putting: A new window on the cognitive mechanisms underlying quiet eye. Sport, Exercise, and Performance Psychology. 2019 Feb;8(1):53. 10.1037/spy0000148.

[32] Carnegie E, Marchant D, Towers S, Ellison P. Beyond visual fixations and gaze behaviour. Using pupillometry to examine the mechanisms in the planning and motor performance of a golf putt. Human Movement Science. 2020 Jun 1;71:102622. doi: 10.1016/j.humov.2020.102622 32452439

[33] Runswick OR, Jewiss M, Sharpe BT, North JS. Context affects Quiet Eye duration and motor performance independent of cognitive effort. Journal of Sport and Exercise Psychology. 2021 Mar 17;43(2):191–7. doi: 10.1123/jsep.2020-0026 33730694

[34] Galanis E, Nurkse L, Kooijman J, Papagiannis E, Karathanasi A, Comoutos N, et al. Effects of a strategic self-talk intervention on attention functions and performance in a golf task under conditions of ego depletion. Sustainability. 2022 Jun 9;14(12):7046. 10.3390/su14127046.

[35] Nagashima Y, Ehara K, Ehara Y, Mitsume A, Kubo K, Mineo S. Effects of continuous carbohydrate intake with gummies during the golf round on interstitial glucose, golf performance, and cognitive performance of competitive golfers: a randomized repeated-measures crossover design. Nutrients. 2023 Jul 21;15(14):3245. doi: 10.3390/nu15143245 37513663 PMC10384188

[36] Filaire E, Sagnol M, Ferrand C, Maso F, Lac G. Psychophysiological stress in judo athletes during competitions. Journal of Sports Medicine and Physical Fitness. 2001 Jun 1;41(2):263–8. 11447372

[37] Mizuno K, Tanaka M, Yamaguti K, Kajimoto O, Kuratsune H, Watanabe Y. Mental fatigue caused by prolonged cognitive load associated with sympathetic hyperactivity. Behavioral and brain functions. 2011 Dec;7:1–7. 10.1186/1744-9081-7-17. 21605411 PMC3113724

[38] Kennedy DO, Scholey AB. A glucose-caffeine ‘energy drink’ameliorates subjective and performance deficits during prolonged cognitive demand. Appetite. 2004 Jun 1;42(3):331–3. doi: 10.1016/j.appet.2004.03.001 15183925

[39] Ataka S, Tanaka M, Nozaki S, Mizuma H, Mizuno K, Tahara T, et al. Effects of oral administration of caffeine and D-ribose on mental fatigue. Nutrition. 2008 Mar 1;24(3):233–8. doi: 10.1016/j.nut.2007.12.002 18178380

[40] Judelson DA, Preston AG, Miller DL, Muñoz CX, Kellogg MD, Lieberman HR. Effects of theobromine and caffeine on mood and vigilance. Journal of clinical psychopharmacology. 2013 Aug 1;33(4):499–506. doi: 10.1097/JCP.0b013e3182905d24 23764688

[41] Lorenzo Calvo J, Fei X, Domínguez R, Pareja-Galeano H. Caffeine and cognitive functions in sports: a systematic review and meta-analysis. Nutrients. 2021 Mar 6;13(3):868. doi: 10.3390/nu13030868 33800853 PMC8000732

