# Supplementary material for: Mental fatigue in golf: A systematic review
Source: PLoS One. 2025 Feb 20;20(2):e0310403. doi: 10.1371/journal.pone.0310403 (PMC11841881; doi:10.1371/journal.pone.0310403)
Supplement: S1 Table — (PDF) [file pone.0310403.s001.pdf]

S1 Table. Detailed search strategy.

Database	Strategy	Results
Web of Science (2009 – Jan 2024)	(ALL=(("mental fatigue" OR "cognitive fatigue" OR "mental effort" OR "cognitive effort" OR "mental exertion" OR "ego depletion"))) AND ALL=(golf)	10
PubMed (2009 – Jan 2024)	("mental fatigue"[All Fields] OR "cognitive fatigue"[All Fields] OR "mental effort"[All Fields] OR "cognitive effort"[All Fields] OR "mental exertion"[All Fields] OR "ego depletion"[All Fields]) AND ("golf"[MeSH Terms] OR "golf"[All Fields])	7
SPORTDiscus (1983 – Jan 2024)	("mental fatigue" OR "cognitive fatigue" OR "mental effort" OR "cognitive effort" OR "mental exertion" OR "ego depletion") AND golf	357
SCOPUS (1994 – Jan 2024)	(ALL ("mental fatigue" OR "cognitive fatigue" OR "mental effort" OR "cognitive effort" OR "mental exertion" OR "ego depletion") AND ALL (golf))	331
CNKI (2019 – Jan 2024)	TKA="Mental Fatigue" OR "Cognitive Fatigue" OR "Mental Effort" OR "Cognitive Effort" OR "Mental Depletion" OR "Ego Depletion" AND TKA="Golf"	2
