# Supplementary material for: Mental fatigue in golf: A systematic review
Source: PLoS One. 2025 Feb 20;20(2):e0310403. doi: 10.1371/journal.pone.0310403 (PMC11841881; doi:10.1371/journal.pone.0310403)
Supplement: S2 Table — (PDF) [file pone.0310403.s002.pdf]

Supplementary information

1. Numbered table of all studies

No.	Author(s)	Year	Title	Journal	Included in Analysis	Reason for Exclusion
1	N. W. Van Yperen	2009	Why some make it and others do not: Identifying psychological factors that predict career success in professional adult soccer	Sport Psychologist	No	Duplicate records
2	P. Gaudreau; A. Nicholls; A. R. Levy	2010	The ups and downs of coping and sport achievement: An episodic process analysis of within-person associations	Journal of Sport and Exercise Psychology	No	Duplicate records
3	A. Piras; M. Timmis; A. Trofè; M. Raffi	2021	Understanding the underlying mechanisms of Quiet Eye: The role of microsaccades, small saccades and pupil-size before final movement initiation in a soccer penalty kick	European Journal of Sport Science	No	Duplicate records
4	L. Resnik; S. L. Klinger; K. Korp; L. S. Walters	2014	TRAINING PROTOCOL FOR POWERED SHOULDER PROSTHESIS	Journal of Rehabilitation Research & Development	No	Duplicate records
5		2012	Sport and Exercise Psychology	Journal of Sport & Exercise Psychology	No	Duplicate records
6		2012	SPORT AND EXERCISE PSYCHOLOGY	Journal of Sport & Exercise Psychology	No	Duplicate records
7		2011	Sport and Exercise Psychology	Journal of Sport & Exercise Psychology	No	Duplicate records
8		2010	SPORT AND EXERCISE PSYCHOLOGY	Journal of Sport & Exercise Psychology	No	Duplicate records
9	M. Ćosić; V. Koprivica	2010	SPECIFICS OF TRAINING STRUCTURE FOR VISUALLY IMPAIRED ATHLETES	Proceedings of the Faculty of Physical Education, University of Banja Luka	No	Duplicate records

10	R. Sides; G. Chow; G. Tenenbaum	2017	Shifts in adaptation: The effects of self-efficacy and task difficulty perception	Journal of Clinical Sport Psychology	No	Duplicate records
11	E. D. V. Dyke; J. L. V. Raalte; E. M. Mullin; B. W. Brewer	2018	Self-Talk and Competitive Balance Beam Performance	Sport Psychologist	No	Duplicate records
12	R.-N. M. Kassim; M. A. Asbullah; M. H. Basal; M. N. Ab Aziz; H. Che Mat; N. S. Nawai; B. S. Zaman	2022	Scrutinizing the Behaviour and Commitment of a Mixed Martial Art Fighting Online Gamer. / Ocena zachowań i zaangażowania graczy uprawiających elektroniczne mieszane sztuki walki	Ido Movement for Culture. Journal of Martial Arts Anthropology	No	Duplicate records
13	N. Stanger; R. Chettle; J. Whittle; J. Poolton	2018	The role of preperformance and in-game emotions in cognitive interference during sport performance: The moderating role of self-confidence and reappraisal	Sport Psychologist	No	Duplicate records
14	N. Konttinen; H. Lyytinen; J. Viitasalo	1998	Rifle-balancing in precision shooting : behavioral aspects and psychophysiological implication (Stabilisation de la carabine de tir de precision = aspects comportementaux et implication psychophysiologique)	Scandinavian Journal of Medicine & Science in Sports	No	Duplicate records
15	K. Berestetska	2019	The Relationship Between Perceived Coaching Behaviors, Intrinsic Motivation, and Scholarship Status on NCAA Division I Tennis Players' Sport Commitment	Journal of Sport Behavior	No	Duplicate records
16	M. Rendell; R. W. Masters; D. Farrow; T. Morris	2011	An Implicit Basis for the Retention Benefits of Random Practice	Journal of Motor Behavior	No	Duplicate records
17	S. A. Kay; L. R. Grimm	2017	Regulatory fit improves fitness for people with low exercise experience	Journal of Sport and Exercise Psychology	No	Duplicate records
18	J. Hardy; N. Comoutos; A. Hatzigeorgiadis	2018	Reflections on the maturing research literature of self-talk in sport: Contextualizing the special issue	Sport Psychologist	No	Duplicate records
19	M. J. Campbell; A. P. Moran; N. Bargary; S. Surmon; L. Bressan; I. C. Kenny	2019	Pupillometry during golf putting: A new window on the cognitive mechanisms underlying quiet eye	Sport, Exercise, and Performance Psychology	No	Duplicate records
20	M. E. Kite; B. E. Whitley	2016	Psychology of Prejudice and Discrimination, Third Edition	Psychology of Prejudice and	No	Duplicate records

				Discrimination, Third Edition		
21	J. M. Porter; T. Beckerman	2016	PRACTICING WITH GRADUAL INCREASES IN CONTEXTUAL INTERFERENCE ENHANCES VISUOMOTOR LEARNING	Kinesiology	No	Duplicate records
22	C. Swann; L. Crust; P. Jackman; S. A. Vella; M. S. Allen; R. Keegan	2017	Performing under pressure: Exploring the psychological state underlying clutch performance in sport	Journal of Sports Sciences	No	Duplicate records
23		2016	Peer-Reviewed Abstracts	Research Quarterly for Exercise & Sport	No	Duplicate records
24	R. G. Cowden; E. L. Worthington Jr	2019	Overcoming failure in sport: A self-forgiveness framework	Journal of Human Sport & Exercise	No	Duplicate records
25	O. Sollie; K. Holmsen; C. Steinbo; Y. Ommundsen; T. Losnegard	2021	Observational vs coaching feedback on non-dominant whole-body motor skill performance -- application to technique training	Scandinavian Journal of Medicine & Science in Sports	No	Duplicate records
26		2017	North American Society for the Psychology of Sport and Physical Activity: San Diego, California June 5-8, 2017	Journal of Sport & Exercise Psychology	No	Duplicate records
27		2020	North American Society for the Psychology of Sport and Physical Activity Virtual Conference June 11–12, 2020	Journal of Sport & Exercise Psychology	No	Duplicate records
28		2021	North American Society for the Psychology of Sport and Physical Activity Virtual Conference June 9-11, 2021	Journal of Sport & Exercise Psychology	No	Duplicate records
29		2019	North American Society for the Psychology of Sport and Physical Activity	Journal of Sport & Exercise Psychology	No	Duplicate records
30		2018	North American Society for the Psychology of Sport and Physical Activity	Journal of Sport & Exercise Psychology	No	Duplicate records
31	D. G.	2023	Multifractal Nonlinearity Moderates	Perceptual	No	Duplicate

	Kelty-Stephen; J. Lee; K. R. Cole; R. K. Shields; M. Mangalam		Feedforward and Feedback Responses to Suprapostural Perturbations	and Motor Skills		records
32		2011	Motor Learning and Control	Journal of Sport & Exercise Psychology	No	Duplicate records
33		2010	MOTOR LEARNING AND CONTROL	Journal of Sport & Exercise Psychology	No	Duplicate records
34		2009	Motor Learning and Control	Journal of Sport & Exercise Psychology	No	Duplicate records
35		2008	Motor Learning and Control	Journal of Sport & Exercise Psychology	No	Duplicate records
36	C. Bédard Thom; F. Guay; C. Trottier	2021	Mental toughness in sport: The Goal-Expectancy-Self-Control (GES) model	Journal of Applied Sport Psychology	No	Duplicate records
37	M. Khojasteh Moghani; R. Zeidabadi; M. R. Shahabi Kaseb; I. Bahreini Borujeni	2021	Mental Fatigue Reduces the Benefits of Self-Controlled Feedback on Learning a Force Production Task	Perceptual and Motor Skills	No	Duplicate records
38	J. T. Patterson; A. Hart; S. Hansen; M. J. Carter; D. Ditor	2016	Measuring investment in learning: Can electrocardiogram provide an indication of cognitive effort during learning?	Perceptual and Motor Skills	No	Duplicate records
39	W. K. Lam; J. X. Fan; Y. Zheng; W. C. C. Lee	2019	Joint and plantar loading in table tennis topspin forehand with different footwork	European Journal of Sport Science	No	Duplicate records
40	T. Devonport; A. Lane; C. L. Fullerton	2016	Introducing sport psychology interventions: Self-control implications	Sport Psychologist	No	Duplicate records
41	H. J. Carson; D. Collins; J. Richards	2014	Intra-individual movement variability during skill transitions: A useful marker?	European Journal of Sport Science	No	Duplicate records
42	H. J. Carson; D. Collins; J. Richards	2014	Intra-individual movement variability during skill transitions: A useful marker?	EUROPEAN JOURNAL OF SPORT SCIENCE	No	Duplicate records

43	H. J. Carson; D. Collins; J. Richards	2014	Intra-individual movement variability during skill transitions: a useful marker?	Eur J Sport Sci	No	Duplicate records
44	N. S. Guest; T. A. VanDusseldorp; M. T. Nelson; J. Grgic; B. J. Schoenfeld; N. D. M. Jenkins; S. M. Arent; J. Antonio; J. R. Stout; E. T. Trexler; A. E. Smith-Ryan; E. R. Goldstein; D. S. Kalman; B. I. Campbell	2021	International society of sports nutrition position stand: caffeine and exercise performance	Journal of the International Society of Sports Nutrition	No	Duplicate records
45	H. J. Carson; D. Collins; J. Richards	2016	Initiating technical refinements in high-level golfers: Evidence for contradictory procedures	European Journal of Sport Science	No	Duplicate records
46	H. J. Carson; D. Collins; J. Richards	2016	Initiating technical refinements in high-level golfers: Evidence for contradictory procedures	Eur J Sport Sci	No	Duplicate records
47	A. Lola; A. Koutsomarkou; G. Tzetzis	2022	Influence of different focus of attention instructions on learning volleyball skills for young novices	Journal of Human Sport and Exercise	No	Duplicate records
48	R. Hayman; R. Polman; E. Borkoles; J. Taylor	2013	The influence of a deliberate practice intervention on the putting performance and subsequent practice behaviours of aspiring elite adolescent golfers	Talent Development and Excellence	No	Duplicate records
49	M. A. Rendell; R. S. W. Masters; D. Farrow; T. Morris	2011	An implicit basis for the retention benefits of random practice	Journal of Motor Behavior	No	Duplicate records
50	D. A. Rosenbaum	2009	Human Motor Control, Second Edition	Human Motor Control, Second Edition	No	Duplicate records
51	J. D. Graham; B. Zhang; D. M. Y. Brown; J. Cairney	2022	The Home Advantage in the National Basketball Association Conference Finals and Finals Series From 1979 to 2019: A Mediation Analysis of Offensive and Defensive Skills	Journal of Sport and Exercise Psychology	No	Duplicate records
52	A. Oliver; P. J. McCarthy; L. Burns	2020	A grounded-theory study of meta-attention in golfers	Sport Psychologist	No	Duplicate records
53	A. Oliver; P. J. McCarthy; L.	2020	A Grounded-Theory Study of Meta-Attention in Golfers	SPORT PSYCHOLO	No	Duplicate records

	Burns			GIST		
54	C. M. Meira; C. O. Cortes; D. M. Corbetta; D. L. Oliveira; S. T. Rodrigues; B. V. F. Silva; M. Massa	2022	Gaze behaviour differentiates elite from non-elite female soccer players: a 2D video projections exploratory study	European Journal of Human Movement	No	Duplicate records
55	G. Ziv; R. Lidor	2019	Gaze Behavior in Golf Putting - A Review	International Journal of Golf Science	No	Duplicate records
56		2016	Full Issue PDF, Volume 87, Supplement 2	Research Quarterly for Exercise & Sport	No	Duplicate records
57	E. Galanis; A. Hatzigeorgiadis; N. Comoutos; F. Charachousi; X. Sanchez	2018	From the lab to the field: Effects of self-talk on task performance under distracting conditions	Sport Psychologist	No	Duplicate records
58	P. Sheeran; T. L. Webb	2012	From Goals to Action	Goal-Directed Behavior	No	Duplicate records
59		2004	FREE COMMUNICATIONS	Journal of Sport & Exercise Psychology	No	Duplicate records
60	K. M. Fisher; J. L. Etnier	2014	Examining the Time Course of Attention During Golf Putts of Two Different Lengths in Experienced Golfers	Journal of Applied Sport Psychology	No	Duplicate records
61	J. G. Cremades	2016	Electro-cortical measures during visual and kinesthetic imagery performance following visual- and auditory-guided instructions	International Journal of Sport and Exercise Psychology	No	Duplicate records
62	M. Shin; Y. Kim; S. Park	2019	Effects of State Anxiety and Ego Depletion on Performance Change in Golf Putting: A Hierarchical Linear Model Application	Perceptual and Motor Skills	No	Duplicate records
63	M. Shin; Y. Kim; S. Park	2019	Effects of State Anxiety and Ego Depletion on Performance Change in Golf Putting: A Hierarchical Linear Model Application	PERCEPTUAL AND MOTOR SKILLS	No	Duplicate records
64	M. Shin; Y. Kim; S. Park	2019	Effects of State Anxiety and Ego Depletion on Performance Change in Golf Putting: A Hierarchical Linear Model Application	Percept Mot Skills	No	Duplicate records
65	L. Filipas; S.	2021	Effects of mental fatigue on soccer-specific	Science and	No	Duplicate

	Borghi; A. La Torre; M. R. Smith		performance in young players	Medicine in Football		records
66		2011	The effects of IQPLUS Focus on cognitive function, mood and endocrine response before and following acute exercise	Journal of the International Society of Sports Nutrition	No	Duplicate records
67	A. Cooke; M. Kavussanu; D. McIntyre; I. D. Boardley; C. Ring	2011	Effects of competitive pressure on expert performance: Underlying psychological, physiological, and kinematic mechanisms	Psychophysio logy	No	Duplicate records
68	T. Simpson; P. Ellison; D. Marchant; E. Carnegie	2022	Effects of Attentional Strategies on Novice Dart Throwing, Quiet Eye Duration and Pupillary Responses	Journal of Motor Behavior	No	Duplicate records
69	R. Mullen; L. Hardy; A. Tattersall	2005	The effects of anxiety on motor performance: A test of the conscious processing hypothesis	Journal of Sport and Exercise Psychology	No	Duplicate records
70	E. Galanis; L. Nurkse; J. Kooijman; E. Papagiannis; A. Karathanasi; N. Comoutos; Y. Theodorakis; A. Hatzigeorgiadis	2022	Effects of a Strategic Self-Talk Intervention on Attention Functions and Performance in a Golf Task under Conditions of Ego Depletion	SUSTAINAB ILITY	No	Duplicate records
71	E. Galanis; A. Hatzigeorgiadis; N. Comoutos; A. Papaioannou; I. D. Morres; Y. Theodorakis	2022	Effects of a strategic self-talk intervention on attention functions	International Journal of Sport and Exercise Psychology	No	Duplicate records
72	T. N. Ziegenfuss; S. M. Habowski; R. Lemieux; J. E. Sandrock; A. W. Kedia; C. M. Kerksick; H. L. Lopez	2015	Effects of a dietary supplement on golf drive distance and functional indices of golf performance	Journal of the International Society of Sports Nutrition	No	Duplicate records
73	A. Whitehead; C. Montgomery; L. Swettenham; N. J. Robinson	2022	The Effect of Think Aloud on Performance and Brain Oxygenation During Cycling – An Exploratory Study	Perceptual and Motor Skills	No	Duplicate records

74	E. J. Stevenson; P. R. Hayes; S. J. Allison	2009	The effect of a carbohydrate-caffeine sports drink on simulated golf performance	Applied Physiology, Nutrition and Metabolism	No	Duplicate records
75	E. J. Stevenson; P. R. Hayes; S. J. Allison	2009	The effect of a carbohydrate-caffeine sports drink on simulated golf performance	APPLIED PHYSIOLOGY NUTRITION AND METABOLISM-PHYSIOLOGIE APPLIQUEE NUTRITION ET METABOLISME	No	Duplicate records
76	E. J. Stevenson; P. R. Hayes; S. J. Allison	2009	The effect of a carbohydrate-caffeine sports drink on simulated golf performance	Appl Physiol Nutr Metab	No	Duplicate records
77	A. Guillot; C. Collet	2005	Duration of mentally simulated movement: A review	Journal of Motor Behavior	No	Duplicate records
78	R. Hayman; R. Polman; J. Taylor; B. Hemmings; E. Borkoles	2011	Development of Elite Adolescent Golfers	Talent Development & Excellence	No	Duplicate records
79	R. Boat; C. Sunderland; S. B. Cooper	2021	Detrimental effects of prior self-control exertion on subsequent sporting skill performance	Scandinavian Journal of Medicine and Science in Sports	No	Duplicate records
80	O. R. Runswick; M. Jewiss; B. T. Sharpe; J. S. North	2021	Context affects quiet eye duration and motor performance independent of cognitive effort	Journal of Sport and Exercise Psychology	No	Duplicate records
81	O. R. Runswick; M. Jewiss; B. T. Sharpe; J. S. North	2021	Context Affects Quiet Eye Duration and Motor Performance Independent of Cognitive Effort	J Sport Exerc Psychol	No	Duplicate records
82	O. R. Runswick; M. Jewiss; B. Sharpe; J. S. North	2021	Context Affects Quiet Eye Duration and Motor Performance Independent of Cognitive Effort	JOURNAL OF SPORT & EXERCISE PSYCHOLOGY	No	Duplicate records

83	D. M. Hill; M. Cheesbrough; P. Gorczynski; N. Matthews	2019	The consequences of choking in sport: A constructive or destructive experience?	Sport Psychologist	No	Duplicate records
84	S. E. Iso-ahola	2015	Conscious versus Nonconscious Mind and Leisure	Leisure Sciences	No	Duplicate records
85	J. Schaefer; S. A. Vella; M. S. Allen; C. A. Magee	2016	Competition Anxiety, Motivation, and Mental Toughness in Golf	Journal of Applied Sport Psychology	No	Duplicate records
86	T. D. Lee; S. P. Swinnen	1994	Cognitive Effort and Motor Learning	Quest (00336297)	No	Duplicate records
87	W. K. Lam; R. S. W. Masters; J. P. Maxwell	2010	Cognitive demands of error processing associated with preparation and execution of a motor skill	Consciousnes s and Cognition	No	Duplicate records
88	W. K. Lam; R. S. W. Masters; J. P. Maxwell	2010	Cognitive demands of error processing associated with preparation and execution of a motor skill	CONSCIOUS NESS AND COGNITION	No	Duplicate records
89	S. A. Wolf; S. Harenberg; K. Tamminen; H. Schmitz	2018	“'Cause You Can't Play This by Yourself”: Athletes' Perceptions of Team Influence on Their Precompetitive Psychological States	Journal of Applied Sport Psychology	No	Duplicate records
90	E. Carnegie; D. Marchant; S. Towers; P. Ellison	2020	Beyond visual fixations and gaze behaviour. Using pupillometry to examine the mechanisms in the planning and motor performance of a golf putt	Human Movement Science	No	Duplicate records
91	E. Carnegie; D. Marchant; S. Towers; P. Ellison	2020	Beyond visual fixations and gaze behaviour. Using pupillometry to examine the mechanisms in the planning and motor performance of a golf putt	HUMAN MOVEMENT SCIENCE	No	Duplicate records
92	J. Vera; R. Molina; D. Cárdenas; B. Redondo; R. Jiménez	2020	Basketball free-throws performance depends on the integrity of binocular vision	European Journal of Sport Science	No	Duplicate records
93	M. Mikicin; M. Kowalczyk	2015	Audio-Visual and Autogenic Relaxation Alter Amplitude of Alpha EEG Band, Causing Improvements in Mental Work Performance in Athletes	APPLIED PSYCHOPH YSIOLOGY AND BIOFEEDBA CK	No	Duplicate records
94	H. O'Shea; A. Moran	2019	Are Fast Complex Movements Unimaginable? Pupillometric Studies of Motor Imagery in Expert Piano Playing	Journal of Motor Behavior	No	Duplicate records
95		2008	Annual Conference of the British Association	Journal of	No	Duplicate

			of Sport and Exercise Sciences 2008	Sports Sciences		records
96		2007	Annual conference of the british association of sport and exercise sciences	Journal of Sports Sciences	No	Duplicate records
97	K. Davies; B. Staples; C. Morris	2020	Accommodate and adapt - Coaching in COVID-19 environment	Coaching & Sport Science Review (Spanish Version)	No	Duplicate records
98		1990	Abstracts of Papers That Will Be Presented at the Thirtieth Annual Meeting of the Society for Psychophysiological Research	Psychophysio logy	No	Duplicate records
99	B. Abernethy; J. P. Maxwell; R. S. W. Masters; J. V. D. Kamp; R. C. Jackson	2012	Attentional Processes in Skill Learning and Expert Performance	Handbook of Sport Psychology: Third Edition	No	Book
100	M. B. Eberly; D. Liu; T. R. Mitchell; T. W. Lee	2013	Attributions and emotions as mediators and/or moderators in the goal-striving process	New Developments in Goal Setting and Task Performance	No	Book
101	G. Martin; J. Pear	2019	Behavior modification: What it is and how to do it	Behavior Modification: What It Is and How To Do It	No	Book
102	R. F. Subotnik; P. Olszewski-Kubili us; F. C. Worrell	2022	Channeling gifted abilities into transformative creative productivity	The Palgrave Handbook of Transformatio nal Giftedness for Education	No	Book
103	H. Richards	2011	Coping and mental toughness	Performance Psychology	No	Book
104	H. Richards	2011	Coping processes in sport	Coping and Emotion in Sport: Second Edition	No	Book
105	M. R. Leary	2007	The Curse of the Self: Self-Awareness, Egotism, and the Quality of Human Life	The Curse of the Self: Self-Awarene ss, Egotism, and the Quality of	No	Book

				Human Life		
106	B. Pageaux; R. Lepers	2018	The effects of mental fatigue on sport-related performance	Progress in Brain Research	No	Book
107	E. Subbotsky	2023	EXAMINING THE PSYCHOLOGICAL FOUNDATIONS OF SCIENCE AND MORALITY: Explaining the Inexplicable	Examining the Psychological Foundations of Science and Morality: Explaining the Inexplicable	No	Book
108	D. Farrow; T. Buszard	2017	Exploring the applicability of the contextual interference effect in sports practice	Progress in Brain Research	No	Book
109	P. Sheeran; T. L. Webb	2012	From goals to action	Goal-Directed Behavior	No	Book
110	D. D. Chen; M. Englar-Carlson	2003	From self-regulation to self-supervision: Lessons from sport psychology to the practice of therapy	Doing Better: Improving Clinical Skills and Professional Competence	No	Book
111	J. O. E. Henderson	2013	Going Far		No	Book
112	C. D. Parks; L. J. Sanna	2018	Group performance and interaction	Group Performance and Interaction	No	Book
113	D. Rosenbaum	2010	Human Motor Control	Human Motor Control	No	Book
114	D. Smith; C. Wakefield	2015	Imagery in sport	Sport and Exercise Psychology: Topics in Applied Psychology: Second Edition	No	Book
115	G. T. Mangine; M. T. Stratton	2019	Incorporating dietary supplements with sports-specific training and competition	Dietary Supplementation in Sport and Exercise: Evidence,	No	Book

				Safety and Ergogenic Benefits		
116	A. Karlinsky; T. N. Welsh; N. J. Hodges	2019	Learning together: Observation and other mechanisms which mediate shared practice contexts	Skill Acquisition in Sport: Research, Theory and Practice	No	Book
117	J. Heckhausen; H. Heckhausen	2008	Motivation and action	Motivation and Action	No	Book
118	C. Englert; I. M. Taylor	2021	Motivation and Self-regulation in Sport and Exercise	Motivation and Self-regulation in Sport and Exercise	No	Book
119	A. Guillot; U. Debarnot; M. Louis; N. Hoyek; C. Collet	2012	Motor imagery and motor performance: Evidence from the sport science literature	The Neurophysiological Foundations of Mental and Motor Imagery	No	Book
120	A. Moran; H. O'Shea	2019	Motor imagery practice and skilled performance in sport From efficacy to mechanisms	Skill Acquisition in Sport: Research, Theory and Practice	No	Book
121	C. A. Coker	2021	Motor Learning and Control for Practitioners, Fifth Edition	Motor Learning and Control for Practitioners, Fifth Edition	No	Book
122	F. Gregory Ashby; V. V. Valentin	2017	Multiple systems of perceptual category learning: Theory and cognitive tests	Handbook of Categorization in Cognitive Science	No	Book
123	S. Gallagher; M. F. Barbe	2022	Musculoskeletal Disorders: The Fatigue Failure Mechanism	Musculoskeletal Disorders: The Fatigue Failure Mechanism	No	Book
124	R. A. Cohen	2014	The Neuropsychology of Attention	The Neuropsychol	No	Book

				ogy of Attention		
125	R. A. Cohen	2013	The neuropsychology of attention: Second edition	The Neuropsychology of Attention: Second Edition	No	Book
126	L. M. Castell; S. J. Stear; L. M. Burke	2015	Nutritional Supplements in Sport, Exercise and Health: An A-Z Guide	Nutritional Supplements in Sport, Exercise and Health: An A-Z Guide	No	Book
127	A. T. Cenkci; M. S. Downing; T. Bircan; K. Perham-Lippman	2023	Overcoming workplace loneliness: Cultivating belonging for a remote workforce	Overcoming Workplace Loneliness: Cultivating Belonging for a Remote Workforce	No	Book
128	R. Venter; R. Grobbelaar	2017	Perceptions and practises of recovery modalities in elite team athletes1	Sport, Recovery, and Performance: Interdisciplinary Insights	No	Book
129	S. Portenga	2021	Performance Skills for Academic Talent Development: Integrating Sport and Performance Psychology Into the Classroom	Talent Development as a Framework for Gifted Education: Implications for Best Practices and Applications in Schools	No	Book
130	J. Perry	2019	Performing Under Pressure: Psychological Strategies for Sporting Success	Performing Under Pressure: Psychological Strategies for Sporting Success	No	Book
131	M. E. Kite; B. E.	2022	Psychology of Prejudice and Discrimination,	Psychology of	No	Book

	Whitley; L. S. Wagner		Fourth Edition	Prejudice and Discrimination, Fourth Edition		
132	J. F. A. Cruz; R. M. Sofia	2016	The pursuit of success and excellence: Self-control in achievement contexts	Psychology of Self-Control: New Research	No	Book
133		2001	References		No	Book
134	S. M. Southwick; D. S. Charney	2018	Resilience: The Science of Mastering Life’s Greatest Challenges	Resilience: The Science of Mastering Life's Greatest Challenges	No	Book
135	R. C. Blagrove; P. R. Hayes	2021	The science and practice of middle and long distance running	The Science and Practice of Middle and Long Distance Running	No	Book
136	J. L. Van Raalte; A. Vincent	2023	SELF- TALK	Routledge Handbook of Applied Sport Psychology: a Comprehensive Guide for Students and Practitioners, Second Edition	No	Book
137	J. B. Vancouver; M. Alicke; L. R. Halper	2017	Self-efficacy	The Self at Work: Fundamental Theory and Research	No	Book
138	C. Cannard; T. Brandmeyer; H. Wahbeh; A. Delorme	2020	Self-health monitoring and wearable neurotechnologies	Handbook of Clinical Neurology	No	Book
139	J. Beckmann; D. Beckmann-Waldenmayer; S. A. Wolf	2023	Self-Regulation in Competitive Sports	Sport and Exercise Psychology: Theory and Application	No	Book
140	C. Stangor	2004	Social groups in action and interaction	Social Groups in Action and	No	Book

				Interaction		
141	C. Stangor	2016	Social groups in action and interaction, Second edition	Social Groups in Action and Interaction, Second Edition	No	Book
142	O. A. Hurley	2018	Sport cyberpsychology	Sport Cyberpsychology	No	Book
143	N. T. Gallucci	2013	Sport psychology: Performance enhancement, performance inhibition, individuals, and teams	Sport Psychology: Performance Enhancement, Performance Inhibition, Individuals, and Teams	No	Book
144	S. Perrey; P. Besson	2018	Studying brain activity in sports performance: Contributions and issues	Progress in Brain Research	No	Book
145	R. T. T. Forman	2019	Towns, ecology, and the land	Towns, Ecology, and the Land	No	Book
146	F. R. Adler; C. J. Tanner	2011	Urban ecosystems: Ecological principles for the built environment	Urban Ecosystems: Ecological Principles for the Built Environment	No	Book
147	R. W. Christina; E. Alpenfels	2012	Why does traditional training fail to optimize playing performance?	Science and Golf IV: Proceedings of the World Scientific Congress of Golf	No	Book
148	E. Galanis; A. Hatzigeorgiadis; N. Zourbanos; Y. Theodorakis	2016	Why Self-Talk Is Effective? Perspectives on Self-Talk Mechanisms in Sport	Sport and Exercise Psychology Research: From Theory to Practice	No	Book
149	E. Triantafyllidis; Z. Li	2021	The Challenges in Modeling Human Performance in 3D Space with Fitts' Law	Conference on Human Factors in	No	Conference Proceedings

				Computing Systems - Proceedings		
150	A. Gaggioli; F. Morganti; A. Meneghini; I. Pozzato; G. Greggio; M. Pigatto; G. Riva	2009	Computer-guided mental practice in neurorehabilitation	Studies in Health Technology and Informatics	No	Conference Proceedings
151	J. M. Williams; V. Krane	1992	Coping styles and self-reported measures of state anxiety and self-confidence	Journal of Applied Sport Psychology	No	Conference Proceedings
152	Q. Guo; J. Wu; B. Li	2015	EEG-based golf putt outcome prediction using support vector machine	IEEE SSCI 2014 - 2014 IEEE Symposium Series on Computational Intelligence - CIBCI 2014: 2014 IEEE Symposium on Computational Intelligence in Brain Computer Interfaces, Proceedings	No	Conference Proceedings
153	S. Janssens; V. Zaytsev	2022	Go with the flow: Software engineers and distractions	Proceedings - ACM/IEEE 25th International Conference on Model Driven Engineering Languages and Systems, MODELS 2022: Companion Proceedings	No	Conference Proceedings
154	G. Wang; A. Suh	2021	A Literature Review on a Neuro-Psychological Approach to Immersive Technology Research	Lecture Notes in Computer	No	Conference Proceedings

				Science (including subseries Lecture Notes in Artificial Intelligence and Lecture Notes in Bioinformatics)		
155	D. E. Aldous; D. S. Loch; R. Prince	2015	Valuing the Australian lifestyle horticulture industry	Acta Horticulturae	No	Conference Proceedings
156	Dapeng Zhu	2023	Humble leadership and athletes' satisfaction with training and competition: chain-mediated effects of coach leadership behavior and coach-athlete relationships	Abstract Proceedings of the 13th National Convention on Sport Science of China	No	Conference Proceedings
157		1997	My 3 good habits to speed play	Golf Digest	No	Magazines
158	F. Brady	1997	Contextual Interference and the Teaching of Golf Skills		No	Title abstract or keywords
159	S. T. Cotterill; R. Sanders; D. Collins	2010	Developing Effective Pre-performance Routines in Golf: Why Don't We Ask the Golfer?	JOURNAL OF APPLIED SPORT PSYCHOLOGY	No	Title abstract or keywords
160	M. Shafizadeh; T. McMorris; J. Sproule	2011	Effect of different external attention of focus instruction on learning of golf putting skill	Percept Mot Skills	No	Title abstract or keywords
161	J. J. Bell; J. Hardy	2009	Effects of Attentional Focus on Skilled Performance in Golf	JOURNAL OF APPLIED SPORT PSYCHOLOGY	No	Title abstract or keywords
162	D. V. J. Marshall; S. J. Hanrahan; N. Comoutos	2016	The Effects of Self-Talk Cues on the Putting Performance of Golfers Susceptible to Detrimental Putting Performances Under High Pressure Settings	International Journal of Golf Science	No	Title abstract or keywords
163	G. Ziv; M. Ochayon; R. Lidor	2019	Enhanced or diminished expectancies in golf putting – Which actually affects performance?	Psychology of Sport and Exercise	No	Title abstract or keywords
164	A. E. Whitehead; J. A. Taylor; R. C.	2015	Examination of the suitability of collecting in event cognitive processes using Think Aloud	Front Psychol	No	Title abstract or keywords

	Polman		protocol in golf			
165	G. Wulf; J. Su	2007	An external focus of attention enhances golf shot accuracy in beginners and experts	RESEARCH QUARTERLY FOR EXERCISE AND SPORT	No	Title abstract or keywords
166	M. R. Farrally; A. J. Cochran; D. J. Crews; M. J. Hurdzan; R. J. Price; J. T. Snow; P. R. Thomas	2003	Golf science research at the beginning of the twenty-first century	JOURNAL OF SPORTS SCIENCES	No	Title abstract or keywords
167	A. Karlinsky; N. J. Hodges	2019	Manipulations to practice organization of golf putting skills through interleaved matched or mismatched practice with a partner	Human Movement Science	No	Title abstract or keywords
168	A. R. Nicholls; N. L. Holt; R. C. J. Polman; D. W. G. James	2005	Stress and coping among international adolescent golfers	JOURNAL OF APPLIED SPORT PSYCHOLOGY	No	Title abstract or keywords
169	J. Finn	2009	Using Mental Skills to Improve Golfing Performance：A Theory-Based Case Study for Golf Coaches		No	Title abstract or keywords
170		2021	ABSTRACTS	German Journal of Sports Medicine / Deutsche Zeitschrift fur Sportmedizin	No	Title abstract or keywords
171		1993	Abstracts of Papers to be Presented at the Thirty-Third Annual Meeting of the Society for Psychophysiological Research	Psychophysiology	No	Title abstract or keywords
172	K. Davies; B. Staples; C. Morris	2020	Accommodate and adapt - Coaching in COVID-19 environment	Coaching & Sport Science Review	No	Title abstract or keywords
173	A. Puklavec; L. Antekolović; P. Mikulić	2021	Acquisition of the long jump skill using varying feedback	Croatian Journal of Education	No	Title abstract or keywords
174	G. Tenenbaum; A. Lane; R. Lidor; S. Razon; R. Schinke	2015	Adaptation: A Two-Perception Probabilistic Conceptual Framework	Journal of Clinical Sport Psychology	No	Title abstract or keywords
175	R. C. Buckley	2018	Aging adventure athletes assess achievements and alter aspirations to maintain self-esteem	Frontiers in Psychology	No	Title abstract or keywords

176	P. S. Meier	2011	Alcohol marketing research: the need for a new agenda	Addiction	No	Title abstract or keywords
177	J. N. Spring; E. F. Sallard; P. Trabucchi; G. P. Millet; J. Barral	2022	Alterations in spontaneous electrical brain activity after an extreme mountain ultramarathon	Biological Psychology	No	Title abstract or keywords
178	M. Bamberger	2002	The Amazing ANNIKA	Sports Illustrated	No	Title abstract or keywords
179	C. Sampson	2011	The Amazing Lives Of Sarah Bradley	Golf World	No	Title abstract or keywords
180	L. Wing Kai; J. P. Maxwell; R. Masters	2009	Analogy Learning and the Performance Motor Skills Under Pressure	Journal of Sport & Exercise Psychology	No	Title abstract or keywords
181	A. Blazevich; N. Cronin; S. Wells	2005	Annual Conference of the British Association of Sport and Exercise Sciences	Journal of Sports Sciences	No	Title abstract or keywords
182	R. Gorgulu; A. Cooke; T. Woodman	2019	Anxiety and Ironic Errors of Performance: Task Instruction Matters	Journal of Sport & Exercise Psychology	No	Title abstract or keywords
183	C. Englert; A. Bertrams	2012	Anxiety, Ego Depletion, and Sports Performance	Journal of Sport & Exercise Psychology	No	Title abstract or keywords
184	F. C. A. Nogueira; M. G. B. Filho; L. M. Lourenço	2019	Application of izof model for anxiety and self-efficacy in volleyball athletes: A case study	Revista Brasileira de Medicina do Esporte	No	Title abstract or keywords
185	E. Tzormpatzakis; E. Galanis; A. Chaldeaki; A. Hatzigeorgiadis	2022	Application of Strategic Self-Talk: An Experimental Study on the Effects on Shooting Stability and Performance	International Journal of Kinesiology and Sports Science	No	Title abstract or keywords
186	H. O'Shea; A. Moran	2019	Are Fast Complex Movements Unimaginable? Pupillometric Studies of Motor Imagery in Expert Piano Playing	Journal of Motor Behavior	No	Title abstract or keywords
187	N. Dlodlo; M. Dhurup	2013	Are university students living in a world of fantasy? Fantasy football motives among students at a South African university of technology	African Journal for Physical, Health Education, Recreation & Dance	No	Title abstract or keywords
188	M. Kottlow; E.	2011	Artists' advance: Decreased upper alpha power	Brain	No	Title abstract or

	Praeg; C. Luethy; L. Jancke		while drawing in artists compared with non-artists	Topography		keywords
189	A. Decker; V. Richard; J. Cairney; P. Jefferies; N. Houser; P. Aubertin; D. Kriellaars	2022	Assessment of Professional Circus Students' Psychological Characteristics at Four Strategic Timepoints over the Scholastic Year: A Longitudinal Study Using the Stress Process Model	Medical Problems of Performing Artists	No	Title abstract or keywords
190	P. G. Ferreira; H. R. Ferreira; J. M. Facco Stefanello	2017	Association of BRAMS with Physiological Variables during a Maximum Test in High Performance Kayak Athletes	Journal of Exercise Physiology Online	No	Title abstract or keywords
191	P. Kozik; J. T. Enns	2021	Athletics and attention: Bi-directional influences in the lab and on the field	Current Issues in Sport Science	No	Title abstract or keywords
192	R. N. Singer; J. H. Cauraugh; M. Murphy; C. Dapeng; R. Lidor	1991	Attentional Control, Distractors, and Motor Performance	Human Performance	No	Title abstract or keywords
193	E. E. Hessler; P. G. Amazeen	2009	Attentional demands on motor-respiratory coordination	Research Quarterly for Exercise and Sport	No	Title abstract or keywords
194	A. Hill; L. Schücker; N. Hagemann; S. A. Babel; C. MacMahon; B. Strauß	2021	Attentional focusing in running: Implicit focus manipulations reflect the effects for explicit instructions	International Journal of Sport & Exercise Psychology	No	Title abstract or keywords
195	X. Sanchez; T. M. Bampouras	2006	Augmented feedback over a short period of time: Does it improve netball goal-shooting performance?	International Journal of Sport Psychology	No	Title abstract or keywords
196	K. Oishi; T. Maeshima	2004	Autonomic nervous system activities during motor imagery in elite athletes	Journal of Clinical Neurophysiology	No	Title abstract or keywords
197	O. Bolliet; C. Collet; A. Dittmar	2005	Autonomic Nervous System Activity During Actual and Mentally Simulated Preparation for Movement	Applied Psychophysiology & Biofeedback	No	Title abstract or keywords
198	R. Roure; C. Collet; C. Deschaumes-Moli	1998	Autonomic nervous system responses correlate with mental rehearsal in volleyball training	European Journal of Applied	No	Title abstract or keywords

	naro; A. Dittmar; H. Rada; G. Delhomme; E. Vernet-Maury			Physiology and Occupational Physiology		
199	T. Iwatsuki; J. W. Navalta; G. Wulf	2019	Autonomy enhances running efficiency	Journal of Sports Sciences	No	Title abstract or keywords
200	P. McCarthy; M. Wilson; R. Keegan; D. Smith	2012	Back to the future: Three myths about applied consultancy work	Sport & Exercise Psychology Review	No	Title abstract or keywords
201	N. T. Ong; N. J. Hodges	2018	Balancing Our Perceptions of the Efficacy of Success-Based Feedback Manipulations on Motor Learning	Journal of Motor Behavior	No	Title abstract or keywords
202	T. Hideyuki; M. Wataru; I. Masato; H. Tanaka; W. Mizuno; M. Iwami	2016	Ball Throwing Without a Ball: Pantomimed Motor Execution Primes the Imagination That an Object is Traveling the Required Distance	Motor Control	No	Title abstract or keywords
203		2011	BASES supplement abstracts	Journal of Sports Sciences	No	Title abstract or keywords
204	J. Vera; R. Molina; D. Cárdenas; B. Redondo; R. Jiménez	2020	Basketball free-throws performance depends on the integrity of binocular vision	European Journal of Sport Science	No	Title abstract or keywords
205	D. de la Peña	2009	The Beneficial Effects of Anticipating Anxiety-Related Symptoms: An Investigation of Paradoxical-Success Imagery in the Laboratory	Journal of Imagery Research in Sport & Physical Activity	No	Title abstract or keywords
206	P. Krause	2009	The Benefits of Cross-Training	AMAA Journal	No	Title abstract or keywords
207	J. Vinstrup; M. D. Jakobsen; P. Madeleine; L. L. Andersen	2020	Biomechanical load during patient transfer with assistive devices: Cross-sectional study	Ergonomics	No	Title abstract or keywords
208	D. Sacha; L. Simmering; M. Adler	2012	A Birdie in the Hand: Asymmetry in Golf Risk Preferences	International Journal of Golf Science	No	Title abstract or keywords
209	E. Conroy; M. Kowal; A. J.	2021	Boosting: Rank and skill deception in esports	Entertainment Computing	No	Title abstract or keywords

	Toth; M. J. Campbell					
210	Toure; P. Secada	2001	The Boy Who Fell to Earth	Tennis	No	Title abstract or keywords
211	R. S. Soliman; S. Lee; S. Eun; A. Z. Mohamed; J. Lee; E. Lee; M. M. Makary; S. M. Kathy Lee; H. J. Lee; W. S. Choi; K. Park	2017	Brain correlates to facial motor imagery and its somatotopy in the primary motor cortex	NeuroReport	No	Title abstract or keywords
212	B. D. Hatfield	2018	Brain Dynamics and Motor Behavior: A Case for Efficiency and Refinement for Superior Performance	Kinesiology Review	No	Title abstract or keywords
213	Q. Gao; L. Zhang	2023	Brief mindfulness meditation intervention improves attentional control of athletes in virtual reality shooting competition: Evidence from fNIRS and eye tracking	Psychology of Sport and Exercise	No	Title abstract or keywords
214	S. J. Tan; G. Kerr; J. P. Sullivan; J. M. Peake	2019	A Brief Review of the Application of Neuroergonomics in Skilled Cognition During Expert Sports Performance	Frontiers in Human Neuroscience	No	Title abstract or keywords
215	A. I. Thuraisingam; D. F. Levine; J. T. Anderson	2006	Can research in sports and other motor skills help improve endoscopy training?	Gastrointestinal Endoscopy	No	Title abstract or keywords
216	G. K. F. Castanho; E. B. Fontes; H. M. Yoshida; B. M. de Campos; E. L. da Silva; S. Appenzeller; P. T. Fernandes	2015	Carbohydrate vs. Placebo: A fMRI BOLD response during different intensities of motor imagery	Revista Neurociencias	No	Title abstract or keywords
217	S. A. Wolf; S. Harenberg; K. Tamminen; H. Schmitz	2018	Cause You Can't Play This by Yourself": Athletes' Perceptions of Team Influence on Their Precompetitive Psychological States"	Journal of Applied Sport Psychology	No	Title abstract or keywords
218	F. Cross-Villasana; P. Gröpel; F. Ehrlenspiel; J. Beckmann	2018	Central theta amplitude as a negative correlate of performance proficiency in a dynamic visuospatial task	Biological Psychology	No	Title abstract or keywords
219	M. Bigliassi; C. I. Karageorghis; A.	2016	Cerebral mechanisms underlying the effects of music during a fatiguing isometric	Psychophysiology	No	Title abstract or keywords

	V. Nowicky; G. Orgs; M. J. Wright		ankle-dorsiflexion task			
220	D. E. Aldous	2013	Challenges associated with green cities of the future	Acta Horticulturae	No	Title abstract or keywords
221	K. J. Jaquess; L. C. Lo; H. Oh; C. Lu; A. Ginsberg; Y. Y. Tan; K. R. Lohse; M. W. Miller; B. D. Hatfield; R. J. Gentili	2018	Changes in Mental Workload and Motor Performance Throughout Multiple Practice Sessions Under Various Levels of Task Difficulty	Neuroscience	No	Title abstract or keywords
222	A. Gong; J. Liu; L. Lua; G. Wu; C. Jiang; Y. Fu	2019	Characteristic differences between the brain networks of high-level shooting athletes and non-athletes calculated using the phase-locking value algorithm	Biomedical Signal Processing and Control	No	Title abstract or keywords
223	J. K. Parker; G. Lovell	2009	Characteristics Affecting the Use of Imagery: A Youth Sports Academy Study	Journal of Imagery Research in Sport & Physical Activity	No	Title abstract or keywords
224	L. J. Roberts; M. S. Jackson; I. H. Grundy	2019	Choking under pressure: Illuminating the role of distraction and self-focus	International Review of Sport and Exercise Psychology	No	Title abstract or keywords
225	N. Hedayatpour; M. Fathi	2013	Co-activation of the knee joint flexor and extensor muscles during multidirectional perturbations after fatiguing exercise	Medicina dello Sport	No	Title abstract or keywords
226	S.-K. Millar; A. R. H. Oldham; M. Donovan	2011	Coaches' Self-Awareness of Timing, Nature and Intent of Verbal Instructions to Athletes	International Journal of Sports Science & Coaching	No	Title abstract or keywords
227	D. D. Grecic; M. B. Ryan	2018	Coaching Golf -- How skilled are we in 'skill'?	Sport Journal	No	Title abstract or keywords
228	A. Nowell	2012	Cognitive	International Journal of Psychology	No	Title abstract or keywords
229	D. Milne; G. Morrison	2015	Cognitive behavioural intervention for the golf yips: A single-case design	Sport & Exercise Psychology Review	No	Title abstract or keywords

230	S. Potter; R. Brown	2012	Cognitive behavioural therapy and persistent post-concussional symptoms: Integrating conceptual issues and practical aspects in treatment	Neuropsychological Rehabilitation	No	Title abstract or keywords
231	H. Herrebrøden; A. R. Jensenius; T. Espeseth; L. Bishop; J. K. Vuoskoski	2023	Cognitive load causes kinematic changes in both elite and non-elite rowers	Human Movement Science	No	Title abstract or keywords
232	B. McKay; M. F. B. Bacelar; J. O. Parma; M. W. Miller; M. J. Carter	2023	The combination of reporting bias and underpowered study designs has substantially exaggerated the motor learning benefits of self-controlled practice and enhanced expectancies: a meta-analysis	International Review of Sport and Exercise Psychology	No	Title abstract or keywords
233	F. Cuenca-Martínez; L. Suso-Martí; M. Grande-Alonso; A. Paris-Alemany; R. L. Touche	2018	Combining motor imagery with action observation training does not lead to a greater autonomic nervous system response than motor imagery alone during simple and functional movements: A randomized controlled trial	PeerJ	No	Title abstract or keywords
234	M. Oytun; M. Çakıcı; C. Tınazcı; H. U. Yavuz	2021	Comparison of anxiety and narcissism levels of different performance groups in female handball players	Anadolu Psikiyatri Dergisi	No	Title abstract or keywords
235	J. J. Nam; D. H. Han	2020	The comparison of perfectionism and commitment between professional and amateur golfers and the association between perfectionism and commitment in the two groups	International Journal of Environmental Research and Public Health	No	Title abstract or keywords
236	D. Mojtahedi; N. Dagnall; A. Denovan; P. Clough; S. Dewhurst; M. Hillier; K. Papageorgiou; J. Perry	2023	Competition Anxiety in Combat Sports and the Importance of Mental Toughness	Behavioral Sciences	No	Title abstract or keywords
237	J. Schaefer; S. A. Vella; M. S. Allen; C. A. Magee	2016	Competition Anxiety, Motivation, and Mental Toughness in Golf	Journal of Applied Sport Psychology	No	Title abstract or keywords
238	S. Munien; S. S. Nkambule; H. Z.	2015	Conceptualisation and use of green spaces in peri-urban communities: Experiences from	African Journal for	No	Title abstract or keywords

	Buthelezi		Inanda, KwaZulu-Natal, South Africa	Physical, Health Education, Recreation & Dance		
239	D. M. Hill; M. Cheesbrough; P. Gorczynski; N. Matthews	2019	The Consequences of Choking in Sport: A Constructive or Destructive Experience?	Sport Psychologist	No	Title abstract or keywords
240	S. Inouye; T. Chi; L. Bradley	2014	Consumer perceived values of Hawaiian attire: The effects of socio-demographic factors	Journal of Fashion Marketing and Management	No	Title abstract or keywords
241	J. Fritsch; A. T. Latinjak	2021	The Content of Goal-Directed Self-Talk in Volitional Processes	Zeitschrift fur Sportpsychologie	No	Title abstract or keywords
242	M. Beik; H. Taheri; A. S. Kakhki; M. Ghoshuni; D. Fazeli	2022	Contextual Interference Effects on Approach Motivation When Learning Timing Tasks: A Frontal Electroencephalography (EEG) Alpha Asymmetry Study in Older Adults	Perceptual & Motor Skills	No	Title abstract or keywords
243	S. Matsumoto; H. Sakuma	2009	Contingent negative variation under competitive situations	Perceptual and Motor Skills	No	Title abstract or keywords
244	A. Boolani; C. Yager; J. Reid; J. Lackman; M. L. Smith	2023	Correlates of depressive mood among graduate-level allied health students: An exploratory study examining trait energy and fatigue	Journal of American College Health	No	Title abstract or keywords
245	C. Collet; F. Di Rienzo; N. Hoyek; A. Guillot	2013	Corrélats neurophysiologiques de l'imagerie motrice. / Neurophysiological correlates of motor imagery	Movement & Sport Sciences / Science & Motricité	No	Title abstract or keywords
246	B. R. Meagher; K. L. Marsh	2014	The costs of cooperation: Action-specific perception in the context of joint action	Journal of Experimental Psychology: Human Perception and Performance	No	Title abstract or keywords
247	R. B. Kreider; J. R. Stout	2021	Creatine in health and disease	Nutrients	No	Title abstract or keywords
248	N. Goldschmied; P. Furley; R. Bush	2020	Critical review of uniform color effects in sports	International Review of	No	Title abstract or keywords

				Sport and Exercise Psychology		
249	H. Y. Cheng; J. N. Wang	2023	A Cross-Cultural Comparison Study of the Relationships Between Perceived Coaching Behaviors and Student Athletes' Competitive Anxiety	Journal of Teaching in Physical Education	No	Title abstract or keywords
250	C. Unkelbach; D. Memmert	2010	Crowd Noise as a Cue in Referee Decisions Contributes to the Home Advantage	Journal of Sport & Exercise Psychology	No	Title abstract or keywords
251	F. AĞDuman	2023	Curling Sporcularında Sürekli Kaygı Düzeyi ve Problem Çözme Becerilerinin İncelenmesi. / Investigation of Trait Anxiety Levels and Problem-Solving Skills in Curling Athletes	Research in Sport Education & Sciences	No	Title abstract or keywords
252	P. W. Fombelle; C. M. Voorhees; M. R. Jenkins; K. Sidaoui; S. Benoit; T. Gruber; A. Gustafsson; I. Abosag	2020	Customer deviance: A framework, prevention strategies, and opportunities for future research	Journal of Business Research	No	Title abstract or keywords
253	H. McDonald; R. Biscaia; M. Yoshida; J. Conduit; J. P. Doyle	2022	Customer Engagement in Sport: An Updated Review and Research Agenda	Journal of Sport Management	No	Title abstract or keywords
254	M. Naughton; T. Scott; D. Weaving; C. Solomon; S. McLean	2023	Defining and quantifying fatigue in the rugby codes	PLoS ONE	No	Title abstract or keywords
255	A. Casado; B. Hanley; L. M. Ruiz-Pérez	2020	Deliberate practice in training differentiates the best Kenyan and Spanish long-distance runners	European Journal of Sport Science	No	Title abstract or keywords
256	M. E. d. S. Nunes; U. C. Correa; M. G. T. X. de Souza; S. Santos	2021	Descriptive versus prescriptive feedback in the learning of golf putting by older persons	International Journal of Sport & Exercise Psychology	No	Title abstract or keywords
257	J. W. Lee; T. Cavanaugh; S. Alessandri	2019	THE DESIGN SCIENCE BEHIND UNIVERSITY LOGOS: HIDDEN FACTORS BEHIND YOUR ICON	Journal of Contemporary Athletics	No	Title abstract or keywords
258	J. Yongick; T. Hai	2014	Detecting Pod Position Effects in the Context of Multi-Segment Sport Programs:	Sport Marketing	No	Title abstract or keywords

			Implications from Four Super Bowl Broadcasts	Quarterly		
259	R. Boat; C. Sunderland; S. B. Cooper	2021	Detrimental effects of prior self-control exertion on subsequent sporting skill performance	Scandinavian Journal of Medicine & Science in Sports	No	Title abstract or keywords
260	J. Cumming; S. J. Cooley; N. Anuar; M.-C. Kosteli; M. L. Quinton; F. Weibull; S. E. Williams	2017	Developing imagery ability effectively: A guide to layered stimulus response training	Journal of Sport Psychology in Action	No	Title abstract or keywords
261	R. Lidor	2004	Developing metacognitive behaviour in physical education classes:the use of task-pertinent learning strategies	Physical Education & Sport Pedagogy	No	Title abstract or keywords
262	A. Khacharem; B. Zoudji; S. Kalyuga; H. Ripoll	2013	Developing Tactical Skills through the Use of Static and Dynamic Soccer Visualizations: An Expert–Nonexpert Differences Investigation	Journal of Applied Sport Psychology	No	Title abstract or keywords
263	D. Farrow; S. Robertson	2017	Development of a Skill Acquisition Periodisation Framework for High-Performance Sport	Sports Medicine	No	Title abstract or keywords
264	R. Hayman; R. Polman; J. Taylor; B. Hemmings; E. Borkoles	2011	Development of Elite Adolescent Golfers	Talent Development & Excellence	No	Title abstract or keywords
265	T. H. Kim; A. Cruz	2011	Differences in brain activation during motor imagery and action observation of golf putting	Scientific Research and Essays	No	Title abstract or keywords
266	T. H. Kim; A. Cruz; J. H. Ha	2011	Differences in learning facilitatory effect of Motor Imagery and Action Observation of golf putting	Journal of Applied Sciences	No	Title abstract or keywords
267	K. Ryu; Y. Choi; J. Kim; Y. Kim; S. Chio	2016	Differential frontal theta activity during cognitive and motor tasks	Journal of Integrative Neuroscience	No	Title abstract or keywords
268		2007	Discussion	Physical Education & Sport Pedagogy	No	Title abstract or keywords
269	R. F. Baumeister; E. J. Masicampo; K. D. Vohs	2011	Do conscious thoughts cause behavior?	Annual Review of Psychology	No	Title abstract or keywords
270	S. L. Koole; N. B.	2012	Do Demanding Conditions Help or Hurt	Social and	No	Title abstract or

	Jostmann; N. Baumann		Self-Regulation?	Personality Psychology Compass		keywords
271	D. Coate; M. Toomey	2014	Do Professional Golf Tour Caddies Improve Player Scoring?	Journal of Sports Economics	No	Title abstract or keywords
272	T. Heinen	2011	Do static-sport athletes and dynamic-sport athletes differ in their visual focused attention?	Sport Journal	No	Title abstract or keywords
273	J. W. Roberts; G. P. Lawrence; T. N. Welsh; M. R. Wilson	2021	Does high state anxiety exacerbate distractor interference?	Human Movement Science	No	Title abstract or keywords
274	A. J. Toth; E. McNeill; K. Hayes; A. P. Moran; M. Campbell	2020	Does mental practice still enhance performance? A 24 Year follow-up and meta-analytic replication and extension	Psychology of Sport and Exercise	No	Title abstract or keywords
275	W. Miller; S. Jeon; M. Kang; J. S. Song; X. Ye	2021	Does Performance-Related Information Augment the Maximal Isometric Force in the Elbow Flexors?	Applied Psychophysiology & Biofeedback	No	Title abstract or keywords
276	A. J. Picazo-Tadeo; F. González-Gómez; J. Guardiola	2017	Does the crowd matter in refereeing decisions? Evidence from Spanish soccer	International Journal of Sport & Exercise Psychology	No	Title abstract or keywords
277	D. P. Calvillo; A. M. Rutchick	2014	Domain knowledge and hindsight bias among poker players	Journal of Behavioral Decision Making	No	Title abstract or keywords
278	G. Gallicchio; C. Ring	2019	Don't look, don't think, just do it! Toward an understanding of alpha gating in a discrete aiming task	Psychophysiology	No	Title abstract or keywords
279	R. Frömer; B. Stürmer; W. Sommer	2016	(Don't) Mind the effort: Effects of contextual interference on ERP indicators of motor preparation	Psychophysiology	No	Title abstract or keywords
280	T. Gabbett; B. Abernethy	2012	Dual-task assessment of a sporting skill: influence of task complexity and relationship with competitive performances	Journal of Sports Sciences	No	Title abstract or keywords
281	A. Guillot; C. Collet	2005	Duration of Mentally Simulated Movement: A Review	Journal of Motor Behavior	No	Title abstract or keywords
282	A. Neal; T. Ballard; J. B. Vancouver	2017	Dynamic Self-Regulation and Multiple-Goal Pursuit	Annual Review of Organizationa	No	Title abstract or keywords

				l Psychology and Organizational Behavior		
283	R. Genoe; C. Kulczycki; H. Marston; S. Freeman; C. Musselwhite; H. Rutherford	2018	E-Leisure and Older Adults: Findings from an International Exploratory Study	Therapeutic Recreation Journal	No	Title abstract or keywords
284	S. Kalyuga; R. Rikers; F. Paas	2012	Educational Implications of Expertise Reversal Effects in Learning and Performance of Complex Cognitive and Sensorimotor Skills	Educational Psychology Review	No	Title abstract or keywords
285	A. Amorim; B. Travassos; D. Monteiro; L. Baptista; P. Duarte-Mendes	2022	Efectos de un programa de visualizatión mental en el rendimiento de atletas de Boccia federados y no federados. / Effects of an imagery programme on the performance of federated and non-federated Boccia athletes	Cuadernos de Psicología del Deporte	No	Title abstract or keywords
286	D. Wang; T. Hu; R. Luo; Q. Shen; Y. Wang; X. Li; J. Qiao; L. Zhu; L. Cui; H. Yin	2022	Effect of Cognitive Reappraisal on Archery Performance of Elite Athletes: The Mediating Effects of Sport-Confidence and Attention	Frontiers in Psychology	No	Title abstract or keywords
287	S. De Groot; J. C. F. De Winter; J. M. L. García; M. Mulder; P. A. Wieringa	2011	The effect of concurrent bandwidth feedback on learning the lane-keeping task in a driving simulator	Human Factors	No	Title abstract or keywords
288	M. Jalalvand; A. Bahram; A. Daneshfar; S. Arsham	2019	The Effect of Gradual Self-Control of Task Difficulty and Feedback on Learning Golf Putting	Research Quarterly for Exercise & Sport	No	Title abstract or keywords
289	H. T. Wang; Y. S. Chen; G. Rekik; C. C. Yang; M. S. Lai; H. L. Tai	2022	The effect of listening to preferred music after a stressful task on performance and psychophysiological responses in collegiate golfers	PeerJ	No	Title abstract or keywords
290	U. Tugtekin; H. F. Odabasi	2023	Effect of multitasking and task characteristics interaction on cognitive load and learning outcomes in virtual reality learning environments	Education and Information Technologies	No	Title abstract or keywords
291	K. Steinhauer; K. Eichhorn	2023	Effect of Practice Structure and Feedback Frequency on Voice Motor Learning in Older Adults	Journal of Voice	No	Title abstract or keywords
292	J. Jimenez-Diaz; K. Chaves-Castro;	2021	Effect of Self-Controlled and Regulated Feedback on Motor Skill Performance and	Journal of Motor	No	Title abstract or keywords

	M. Morera-Castro		Learning: A Meta-Analytic Study	Behavior		
293	K. J. Jaquess; Y. Lu; A. Ginsberg; S. Kahl Jr; C. Lu; B. Ritland; R. J. Gentili; B. D. Hatfield	2021	Effect of Self-Controlled Practice on Neuro-Cortical Dynamics During the Processing of Visual Performance Feedback	Journal of Motor Behavior	No	Title abstract or keywords
294	A. Whitehead; C. Montgomery; L. Swettenham; N. J. Robinson	2022	The Effect of Think Aloud on Performance and Brain Oxygenation During Cycling – An Exploratory Study	Perceptual & Motor Skills	No	Title abstract or keywords
295	F. Tavares; P. Healey; T. B. Smith; M. Driller	2017	EFFECT OF TRAINING LOAD ON ACUTE FATIGUE AND WELLNESS DURING AN IN-SEASON NON-COMPETITIVE WEEK IN ELITE RUGBY ATHLETES	New Zealand Journal of Sports Medicine	No	Title abstract or keywords
296	H. Singh; A. Gokeler; A. Benjaminse	2021	Effective Attentional Focus Strategies after Anterior Cruciate Ligament Reconstruction: A Commentary	International Journal of Sports Physical Therapy	No	Title abstract or keywords
297	J. Kim; I. Jo; Y. Ma; H. Yoon; D. Yook	2023	Effective Modeling on Learning Ballet Online	Education Sciences	No	Title abstract or keywords
298	T. N. Ziegenfuss; S. M. Habowski; R. Lemieux; J. E. Sandrock; A. W. Kedia; C. M. Kerksick; H. L. Lopez	2015	Effects of a dietary supplement on golf drive distance and functional indices of golf performance	Journal of the International Society of Sports Nutrition	No	Title abstract or keywords
299	P. Bustillo-Casero; S. Cebrian-Bou; C. Cruz-Montecinos; A. Pardo; X. García-Massó	2020	Effects of A Dual-Task Intervention in Postural Control and Cognitive Performance in Adolescents	Journal of Motor Behavior	No	Title abstract or keywords
300	T. D. Patrick; D. W. Hrycaiko	1998	Effects of a mental training package on an endurance performance	Sport Psychologist	No	Title abstract or keywords
301	P. Post; G. Young; D. Simpson	2018	The Effects of a PETTLEP Imagery Intervention on Learners' Coinoident Anticipation Timing Performance	Journal of Applied Sport Psychology	No	Title abstract or keywords
302	E. Galanis; A. Hatzigeorgiadis; N. Comoutos; A.	2022	Effects of a strategic self-talk intervention on attention functions	International Journal of Sport &	No	Title abstract or keywords

	Papaioannou; I. D. Morres; Y. Theodorakis			Exercise Psychology		
303	R. Mullen; L. Hardy; A. Tattersall	2005	The Effects of Anxiety on Motor Performance: A Test of the Conscious Processing Hypothesis	Journal of Sport & Exercise Psychology	No	Title abstract or keywords
304	A. Asadi; M. R. Saeedpour-Parizi; C. A. Aiken; Z. Jahanbani; D. Houminiyan Sharif Abadi; T. Simpson; D. Marchant	2022	Effects of attentional focus and cognitive load on novice dart throwing: Evidence from quiet eye duration and pupillary responses	Human Movement Science	No	Title abstract or keywords
305	T. Simpson; P. Ellison; D. Marchant; E. Carnegie	2022	Effects of Attentional Strategies on Novice Dart Throwing, Quiet Eye Duration and Pupillary Responses	Journal of Motor Behavior	No	Title abstract or keywords
306	R. Gorgulu; E. Gokcek	2021	The Effects of Avoiding Instructions Under Pressure: An Examination of the Volleyball Serving Task	Journal of Human Kinetics	No	Title abstract or keywords
307	K. L. Chambers; J. N. Vickers	2006	Effects of Bandwidth Feedback and Questioning on the Performance of Competitive Swimmers	Sport Psychologist	No	Title abstract or keywords
308	A. M. Ehlert; H. M. Twiddy; P. B. Wilson	2020	The Effects of Caffeine Mouth Rinsing on Exercise Performance: A Systematic Review	International Journal of Sport Nutrition & Exercise Metabolism	No	Title abstract or keywords
309	L. J. Roberts; M. S. Jackson; I. H. Grundy	2021	The effects of cognitive interference during the preparation and execution of the golf swing	International Journal of Sport & Exercise Psychology	No	Title abstract or keywords
310	A. Cooke; M. Kavussanu; D. McIntyre; I. D. Boardley; C. Ring	2011	Effects of competitive pressure on expert performance: Underlying psychological, physiological, and kinematic mechanisms	Psychophysiology	No	Title abstract or keywords
311	S. Szczepan; K. Zatoń; F. Cuenca-Fernández; A. Gay; R. Arellano	2018	The effects of concurrent visual versus verbal feedback on swimming strength task execution	Baltic Journal of Health & Physical Activity	No	Title abstract or keywords

312	S. Kent; T. J. Devonport; A. M. Lane; W. Nicholls; A. P. Friesen	2018	The Effects of Coping Interventions on Ability to Perform Under Pressure	Journal of Sports Science & Medicine	No	Title abstract or keywords
313	Y. He; J. Lu; H. Huang; S. He; N. Ma; Z. Sha; Y. Sun; X. Li	2019	The effects of flipped classrooms on undergraduate pharmaceutical marketing learning: A clustered randomized controlled study	PLoS ONE	No	Title abstract or keywords
314	Z. V. Vleet; A. K C; K. J. Lee; M. Fernandez	2023	The effects of green space on college students' mood	Journal of American College Health	No	Title abstract or keywords
315	H. MacLeod; S. Cooper; S. Bandelow; R. Malcolm; C. Sunderland	2018	Effects of heat stress and dehydration on cognitive function in elite female field hockey players	BMC Sports Science, Medicine & Rehabilitation	No	Title abstract or keywords
316	D. Alder; P. R. Ford; J. Causer; A. M. Williams	2016	The Effects of High- and Low-Anxiety Training on the Anticipation Judgments of Elite Performers	Journal of Sport & Exercise Psychology	No	Title abstract or keywords
317	W. Jang; Y. J. Kim; K. Chang; T. Kim	2022	The effects of high-tech cameras on sports consumers' viewing experiences: the moderating role of sports involvement	International Journal of Sports Marketing and Sponsorship	No	Title abstract or keywords
318	Y. Sarig; M. C. Ruiz; A. Hatzigeorgiadis; G. Tenenbaum	2023	The Effects of Instructional Self-Talk on Quiet-Eye Duration and Golf-Putting Performance	Sport Psychologist	No	Title abstract or keywords
319	T. A. C. Oliveira; R. A. Denardi; G. Tani; U. C. Corrêa	2013	Effects of Internal and External Attentional Foci on Motor Skill Learning: Testing the Automation Hypothesis	Human Movement	No	Title abstract or keywords
320	A. G. Parker; J. Gordon; A. Thornton; A. Byars; J. Lubker; M. Bartlett; M. Byrd; J. Oliver; S. Simbo; C. Rasmussen; M. Greenwood; R. B.	2011	The effects of IQPLUS Focus on cognitive function, mood and endocrine response before and following acute exercise	Journal of the International Society of Sports Nutrition	No	Title abstract or keywords

	Kreider					
321	L. Filipas; S. Borghi; A. La Torre; M. R. Smith	2021	Effects of mental fatigue on soccer-specific performance in young players	Science & Medicine in Football	No	Title abstract or keywords
322	J. Wonseok; K. Yong Jae; S. Stepchenkova	2014	The Effects of Message Appeal on Consumer Attitude Toward Sporting Events	International Journal of Sport Communicati on	No	Title abstract or keywords
323	C. A. Larson; M. R. Surber-Berro	2006	The effects of observational feedback and verbal cues on the motor learning of an aimed reach-and-point task	Pediatric Physical Therapy	No	Title abstract or keywords
324	L. Hardy; A. Hutchinson	2007	Effects of performance anxiety on effort and performance in rock climbing: A test of processing efficiency theory	Anxiety, Stress & Coping	No	Title abstract or keywords
325	R. S. Lindsay; A. R. H. Oldham; E. J. Drinkwater; M. Spittle; A. G. Storey	2022	Effects of personalised motor imagery on the development of a complex weightlifting movement	International Journal of Sport & Exercise Psychology	No	Title abstract or keywords
326	D. J. Brown; D. Fletcher	2017	Effects of Psychological and Psychosocial Interventions on Sport Performance: A Meta-Analysis	Sports Medicine	No	Title abstract or keywords
327	M. S. Januário; L. S. Figueiredo; L. L. Portes; R. N. Benda	2019	Effects of Self-Controlled Knowledge of Results on Learning a Taekwondo Serial Skill	Perceptual & Motor Skills	No	Title abstract or keywords
328	A. J. Davis; B. Crittenden; E. Cohen	2021	Effects of social support on performance outputs and perceived difficulty during physical exercise	Physiology and Behavior	No	Title abstract or keywords
329	M. A. Dhouibi; I. Miladi; G. Racil; S. Hammoudi; J. Coquart	2021	The Effects of Sporting and Physical Practice on Visual and Kinesthetic Motor Imagery Vividness: A Comparative Study Between Athletic, Physically Active, and Exempted Adolescents	Frontiers in Psychology	No	Title abstract or keywords
330	T. Zult; J. Allsop; M. A. Timmis; S. Pardhan	2019	The effects of temporal pressure on obstacle negotiation and gaze behaviour in young adults with simulated vision loss	Scientific Reports	No	Title abstract or keywords
331	M. Zabala; C. Sánchez-Muñoz; M. Mateo	2009	Effects of the administration of feedback on performance of the BMX cycling gate start	Journal of Sports Science & Medicine	No	Title abstract or keywords
332	M. A. Souissi; Y. Elghoul; H.	2023	The Effects of Three Correction Strategies of Errors on the Snatch Technique in	Journal of Strength and	No	Title abstract or keywords

	Souissi; L. Masmoudi; A. Ammar; H. Chtourou; N. Souissi		10-12-Year-Old Children: A Randomized Controlled Trial	Conditioning Research		
333	E. P. Hebert; B. M. Williams	2017	Effects of Three Types of Attentional Focus on Standing Long Jump Performance	Journal of Sport Behavior	No	Title abstract or keywords
334	A. Green; C. Dafkin; S. Kerr; W. McKinon	2015	The effects of walking on golf drive performance in two groups of golfers with different skill levels	Biology of Exercise	No	Title abstract or keywords
335	J. G. Cremades	2016	Electro-cortical measures during visual and kinesthetic imagery performance following visual- and auditory-guided instructions	International Journal of Sport & Exercise Psychology	No	Title abstract or keywords
336	Z. Brabencová; D. Pánek; D. Pavlů; L. Kovářová	2014	Electroencephalographic correlates of the onset of central fatigue during prolonged violin play of professional musicians	Rehabilitace a Fyzikalni Lekarstvi	No	Title abstract or keywords
337	A. Sidhu; A. Cooke	2021	Electroencephalographic neurofeedback training can decrease conscious motor control and increase single and dual-task psychomotor performance	Experimental Brain Research	No	Title abstract or keywords
338	L. Ismail; W. Karwowski; P. A. Hancock; R. Taiar; R. Fernandez-Sumano	2023	Electroencephalography (EEG) Physiological Indices Reflecting Human Physical Performance: A Systematic Review Using Updated PRISMA	Journal of Integrative Neuroscience	No	Title abstract or keywords
339	M. Ghasemian; H. Taheri; A. S. Kakhki; M. Ghoshuni	2017	Electroencephalography Pattern Variations During Motor Skill Acquisition	Perceptual & Motor Skills	No	Title abstract or keywords
340	E. Kemp; S. W. Kopp	2011	Emotion regulation consumption: When feeling better is the aim	Journal of Consumer Behaviour	No	Title abstract or keywords
341	A. D. Tian; J. Schroeder; G. Häubl; J. L. Risen; M. I. Norton; F. Gino	2018	Enacting rituals to improve self-control	Journal of Personality and Social Psychology	No	Title abstract or keywords
342	B. R. T. Roberts; C. M. MacLeod; M. A. Fernandes	2022	The Enactment Effect: A Systematic Review and Meta-Analysis of Behavioral, Neuroimaging, and Patient Studies	Psychological Bulletin	No	Title abstract or keywords
343	P. J. Simmonds;	2023	Enhanced expectancies benefit performance	Human	No	Title abstract or

	C. J. Wakefield; G. Coyles; J. W. Roberts		under distraction, but compromise it under stress: Exploring the OPTIMAL theory	Movement Science		keywords
344	H. Nishizawa; T. Kimura	2017	Enhancement of motor skill learning by a combination of ideal model-observation and self-observation	Journal of Physical Therapy Science	No	Title abstract or keywords
345	C. H. Shea; G. Wulf; C. Whitacre	1999	Enhancing Training Efficiency and Effectiveness Through the Use of Dyad Training	Journal of Motor Behavior	No	Title abstract or keywords
346	S. Alexandru; G. Diana	2010	THE ETIOLOGY OF PAIN AND ABNORMAL ANATOMICAL CHANGES IN THE SPINE -- A LITERATURE REVIEW	Ovidius University Annals, Series Physical Education & Sport/Science, Movement & Health	No	Title abstract or keywords
347	M. El-Kishawi; K. Khalaf; S. A. Kawas	2021	Evaluation of Conscious Monitoring and Movement Control Efforts Among Dental Students	Perceptual & Motor Skills	No	Title abstract or keywords
348	K. Y. Lai; I. Scott; Z. Sun	2019	Everyday Use of the City Cemetery: A Study of Environmental Qualities and Perceived Restorativeness in a Scottish Context	Urban Science	No	Title abstract or keywords
349	M. Jewiss; O. R. Runswick; I. Greenlees	2023	An Examination of the Challenge/Threat State and Sport-Performance Relationship While Controlling for Past Performance	Journal of Sport and Exercise Psychology	No	Title abstract or keywords
350	J. Marmeleira	2013	An examination of the mechanisms underlying the effects of physical activity on brain and cognition	European Reviews of Aging & Physical Activity	No	Title abstract or keywords
351	F. Moen; R. A. Federici; F. Abrahamsen	2015	Examining possible Relationships between mindfulness, stress, school-and sport performances and athlete burnout	International Journal of Coaching Science	No	Title abstract or keywords
352	J. W. Roberts; M. R. Wilson; J. K. Skultety; J. L. Lyons	2018	Examining the effect of state anxiety on compensatory and strategic adjustments in the planning of goal-directed aiming	Acta Psychologica	No	Title abstract or keywords
353	K. M. Fisher; J. L. Etnier	2014	Examining the Time Course of Attention During Golf Putts of Two Different Lengths in Experienced Golfers	Journal of Applied Sport Psychology	No	Title abstract or keywords
354	J. Francisco Filipe	2011	Exercise Can Improve Speed of Behavior in	Journal of	No	Title abstract or

	Marmeleira; F. M. S. de Melo; M. Tlemcani; M. Adriano Bandeira Godinho		Older Drivers	Aging & Physical Activity		keywords
355	S. E. Iso-Ahola	2013	Exercise: Why it is a challenge for both the nonconscious and conscious mind	Review of General Psychology	No	Title abstract or keywords
356	M. Bahmani; A. Bahram; J. A. Diekfuss; S. Arsham	2019	An expert's mind in action: Assessing attentional focus, workload and performance in a dynamic, naturalistic environment	Journal of Sports Sciences	No	Title abstract or keywords
357	N. L. Holt; L. Homan; K. Youngoh; K. Klein	2014	Exploring Experiences of Running an Ultramarathon	Sport Psychologist	No	Title abstract or keywords
358	A. Moran; M. Campbell; J. Toner	2019	Exploring the cognitive mechanisms of expertise in sport: Progress and prospects	Psychology of Sport and Exercise	No	Title abstract or keywords
359	N. J. Hodges; K. R. Lohse; A. Wilson; S. B. Lim; D. Mulligan	2014	Exploring the Dynamic Nature of Contextual Interference: Previous Experience Affects Current Practice But Not Learning	Journal of Motor Behavior	No	Title abstract or keywords
360	J. K. Lee; T. Ahn; K.-Y. Lee	2016	Exploring the impact of country-of-origin fit and team identification in sports brand evaluation	European Sport Management Quarterly	No	Title abstract or keywords
361	B. Evans; M. Eys; S. Wolf	2013	Exploring the Nature of Interpersonal Influence in Elite Individual Sport Teams	Journal of Applied Sport Psychology	No	Title abstract or keywords
362	R. Abdollahipour; W. M. Land; A. Cereser; S. Chiviacowsky	2020	External relative to internal attentional focus enhances motor performance and learning in visually impaired individuals	Disability and Rehabilitation	No	Title abstract or keywords
363	S. Hughes	2021	FACING UP: LIAM LIVINGSTONE	Cricketer	No	Title abstract or keywords
364	J. M. Bootsma; S. R. Caljouw; M. P. Veldman; N. M. Maurits; J. C. Rothwell; T. Hortobágyi	2020	Failure to Engage Neural Plasticity through Practice of a High-difficulty Task is Accompanied by Reduced Motor Skill Retention in Older Adults	Neuroscience	No	Title abstract or keywords
365	I. Halperin; D. W. Chapman; C. Abbiss; K. G.	2019	False-performance feedback does not affect punching forces and pacing of elite boxers	Journal of Sports Sciences	No	Title abstract or keywords

	Thompson					
366	A. J. Amorose; P. J. K. Smith	2003	Feedback as a Source of Physical Information: Effects of Age, Experience and Type of Feedback	Journal of Sport & Exercise Psychology	No	Title abstract or keywords
367	C. Rominger; D. Memmert; I. Papousek; C. M. Perchtold-Stefan; E. M. Weiss; M. Benedek; A. R. Schwerdtfeger; A. Fink	2020	Female and male soccer players recruited different cognitive processes when generating creative soccer moves	Psychology of Sport and Exercise	No	Title abstract or keywords
368	S. Lee; S. S. Byun; J. An; Y. K. Lee; J. Y. Lee; W. K. Shin; J. M. Jang	2020	The Field application of youth soccer training program based on the motor learning techniques	Korean Journal of Sport Science	No	Title abstract or keywords
369	J. Siniscalchi; C. D. Pierskalla	2005	FLYING SAUCERS	Parks & Recreation	No	Title abstract or keywords
370	C. Rumpf; C. Breuer	2018	Focus on Brand Choice: Assessing the Behavioral Response to Sponsorship-Linked Communication	Journal of Sport Management	No	Title abstract or keywords
371	P. Dimeo	2001	Football and Politics in Bengal: Colonialism, Nationalism, Communalism	Soccer & Society	No	Title abstract or keywords
372	N. J. Duru	2019	For Fear of the Fans: An Argument for Holding Sports Teams Accountable for Fans' Post-Match Conduct	Texas Review of Entertainment & Sports Law	No	Title abstract or keywords
373	D. W. Eccles; Y. Balk; T. W. Gretton; N. Harris	2022	The forgotten session": Advancing research and practice concerning the psychology of rest in athletes"	Journal of Applied Sport Psychology	No	Title abstract or keywords
374	S. Qiu; L. Cai	2021	A framework of tourist behavioral investment and application to small city tourism marketing	Asia Pacific Journal of Tourism Research	No	Title abstract or keywords
375		2002	Free Communications	Journal of Sport & Exercise Psychology	No	Title abstract or keywords
376	R. S. Johnson	2007	FROM CUB TO MAN	Men's Fitness	No	Title abstract or keywords
377	E. Galanis; A. Hatzigeorgiadis; N. Comoutos; F.	2018	From the Lab to the Field: Effects of Self-Talk on Task Performance Under Distracting Conditions	Sport Psychologist	No	Title abstract or keywords

	Charachousi; X. Sanchez					
378	M. Lee; G. H. Shin; S. W. Lee	2020	Frontal EEG Asymmetry of Emotion for the Same Auditory Stimulus	IEEE Access	No	Title abstract or keywords
379	K. Shih-Chun; H. Chung-Ju; H. Tsung-Min	2013	Frontal Midline Theta is a Specific Indicator of Optimal Attentional Engagement During Skilled Putting Performance	Journal of Sport & Exercise Psychology	No	Title abstract or keywords
380		2017	Full Issue PDF, Volume 88, Supplement 1	Research Quarterly for Exercise & Sport	No	Title abstract or keywords
381	A. Carolina-Paludo; F. Nunes-Rabelo; M. Maciel-Batista; I. Rúbila-Maciel; M. Peikriszwili-Tartaruga; A. C. Simões	2020	Game location effect on pre-competition cortisol concentration and anxiety state: A case study in a futsal team	Revista de Psicologia del Deporte	No	Title abstract or keywords
382	G. Ziv; R. Lidor	2021	Gaze Behavior in Golf Putting - A Review	International Journal of Golf Science	No	Title abstract or keywords
383	C. M. Meira Jr; C. O. Cortes; D. M. Corbetta; D. L. Oliveira; S. T. Rodrigues; B. V. F. Silva; M. Massa	2022	Gaze behaviour differentiates elite from non-elite female soccer players: a 2D video projections exploratory study	European Journal of Human Movement	No	Title abstract or keywords
384	D. Veličković; D. Radovanović	2018	GENDER DIFFERENCES IN CHESS PERFORMANCE. / RODNE RAZLIKE U ŠAHOVSKIM PERFORMANSAMA	Facta Universitatis: Series Physical Education & Sport	No	Title abstract or keywords
385	Y. Oc; K. Plangger	2022	GIST do it! How motivational mechanisms help wearable users develop healthy habits	Computers in Human Behavior	No	Title abstract or keywords
386	D. Fleming	2022	GOAT@23	Tennis	No	Title abstract or keywords
387	C. Beck	1999	The Golf Club	Successful Meetings	No	Title abstract or keywords
388	D. Higdon	1997	Grass court crash course	Tennis	No	Title abstract or

						keywords
389	A. Skulmowski	2023	Guidelines for Choosing Cognitive Load Measures in Perceptually Rich Environments	Mind, Brain, and Education	No	Title abstract or keywords
390	T. Finkenzeller; M. Doppelmayr; G. Amesberger	2012	Heart rate variability as an indicator of attention focus in golfers of differing performance levels	Zeitschrift fur Sportpsychologie	No	Title abstract or keywords
391	B. Min; I. Choi	2016	Heavy-heartedness biases your weight perception	Journal of Social Psychology	No	Title abstract or keywords
392	B. Daniel; R. Philipp; M. Gareth	2021	Helping athletes flourish using mindfulness and acceptance approaches - an introduction and mini review	SEMS-Journal	No	Title abstract or keywords
393	S. Adams; L. Wilson; S. Lucas; J. Cotter	2017	HIGHER CEREBRAL BLOOD FLOW RESPONSES TO SWIMMING THAN TO LAND-BASED ARM OR LEG EXERCISE	New Zealand Journal of Sports Medicine	No	Title abstract or keywords
394	D. B. King; B. L. Raymond	1995	History of Sport Psychology in Cultural Magazines of the Victorian Era	Sport Psychologist	No	Title abstract or keywords
395	J. D. Graham; B. Zhang; D. M. Y. Brown; J. Cairney	2022	The Home Advantage in the National Basketball Association Conference Finals and Finals Series From 1979 to 2019: A Mediation Analysis of Offensive and Defensive Skills	Journal of Sport & Exercise Psychology	No	Title abstract or keywords
396	M. Oxfeldt; L. B. Dalgaard; A. A. Jørgensen; M. Hansen	2020	Hormonal Contraceptive Use, Menstrual Dysfunctions, and Self-Reported Side Effects in Elite Athletes in Denmark	International Journal of Sports Physiology & Performance	No	Title abstract or keywords
397	D. Aldous	2008	HORTICULTURAL TOURISM IN AUSTRALIA	Australasian Parks & Leisure	No	Title abstract or keywords
398	C. Pocock; N. E. Bezodis; K. Davids; J. S. North	2018	Hot hands, cold feet? Investigating effects of interacting constraints on place kicking performance at the 2015 Rugby Union World Cup	European Journal of Sport Science	No	Title abstract or keywords
399	K. C. Heesch; J. Van Uffelen; W. J. Brown	2014	How Do Older Adults Respond to Active Australia Physical Activity Questions? Lessons From Cognitive Interviews	Journal of Aging & Physical Activity	No	Title abstract or keywords
400	G. Van Sickle	2012	HOW DO YOU LIKE ME NOW?	Sports Illustrated	No	Title abstract or keywords
401	L. Zarantonello; S. Grappi; M. Formisano	2023	How technological and natural consumption experiences impact consumer well-being: The role of consumer mindfulness and fatigue	Psychology and Marketing	No	Title abstract or keywords
402	S. Pineda-Hernández	2022	How to play under pressure: EEG monitoring of mental activation training in a professional	Physiology and Behavior	No	Title abstract or keywords

			tennis player			
403	M. J. Turner	2016	HOW TO SUPPORT YOUR ATHLETES TO THRIVE UNDER PRESSURE BY TIPPING THE BALANCE	Co-Kinetic Journal	No	Title abstract or keywords
404	S. P. J. Goodman; M. A. Immink; F. E. Marino	2022	Hypohydration alters pre-frontal cortex haemodynamics, but does not impair motor learning	Experimental Brain Research	No	Title abstract or keywords
405	S. Zhang; R. Roberts; T. Woodman; A. Cooke	2020	I Am Great, but Only When I Also Want to Dominate: Maladaptive Narcissism Moderates the Relationship Between Adaptive Narcissism and Performance Under Pressure	Journal of Sport & Exercise Psychology	No	Title abstract or keywords
406	C. C. Gonzalez; J. Causer; R. C. Miall; M. J. Grey; G. Humphreys; A. M. Williams	2017	Identifying the causal mechanisms of the quiet eye	European Journal of Sport Science	No	Title abstract or keywords
407	M. Bieleke; W. Wolff; C. Englert; P. M. Gollwitzer	2021	If-Then Planning in Sports: A Scoping Review	Zeitschrift fur Sportpsychologie	No	Title abstract or keywords
408	J. D. Graham; S. R. Bray	2012	Imagery and endurance: Does imagery impair performance by depleting self-control strength?	Journal of Imagery Research in Sport and Physical Activity	No	Title abstract or keywords
409	L. A. Barella; J. L. Etnier; C. Yu-Kai	2010	The Immediate and Delayed Effects of an Acute Bout of Exercise on Cognitive Performance of Healthy Older Adults	Journal of Aging & Physical Activity	No	Title abstract or keywords
410	B. Dwyer	2011	The Impact of Fantasy Football Involvement on Intentions to Watch National Football League Games on Television	International Journal of Sport Communication	No	Title abstract or keywords
411	L. Cesanelli; B. Ylaitė; G. Messina; D. Zangla; S. Cataldi; A. Palma; A. Iovane	2021	The impact of fluid loss and carbohydrate consumption during exercise, on young cyclists’ fatigue perception in relation to training load level	International Journal of Environmental Research and Public Health	No	Title abstract or keywords
412	L. Xiong; X. Huang; J. Li; P. Mao; X. Wang; R. Wang; M. Tang	2018	Impact of indoor physical environment on learning efficiency in different types of tasks: A 3 × 4 × 3 full factorial design analysis	International Journal of Environmental Research and Public	No	Title abstract or keywords

				Health		
413	A. Lohneiss; B. Hill	2014	The impact of processing athlete transgressions on brand image and purchase intent	European Sport Management Quarterly	No	Title abstract or keywords
414	L. Chen; S. Yang	2021	Impact of Sports Wearable Testing Equipment Based on Vision Sensors on the Sports Industry	Journal of Sensors	No	Title abstract or keywords
415	A. Moran; M. Campbell; D. Ranieri	2018	Implications of eye tracking technology for applied sport psychology	Journal of Sport Psychology in Action	No	Title abstract or keywords
416	Y. Chang; Y. J. Ko; B. D. Carlson	2018	Implicit and Explicit Affective Evaluations of Athlete Brands: The Associative Evaluation–Emotional Appraisal–Intention Model of Athlete Endorsements	Journal of Sport Management	No	Title abstract or keywords
417	E. Lundkvist; H. Gustafsson; G. Björklund; P. Davis; A. Ivarsson	2021	Relating Competitive Golfers' Perceived Emotions and Performance	Perceptual and Motor Skills	No	Title abstract or keywords
418	D. de la Peña; N. Murray; C. Janelle	2008	Implicit overcompensation: The influence of negative self-instructions on performance of a self-paced motor task	Journal of Sports Sciences	No	Title abstract or keywords
419	T. Schefke; P. Gronek	2010	IMPROVING ATTENTIONAL PROCESSES IN SPORT: DEFINING ATTENTION, ATTENTIONAL SKILLS AND ATTENTION TYPES	Studies in Physical Culture & Tourism	No	Title abstract or keywords
420	S. D. Benum; F. R. van der Weel; A. L. H. van der Meer	2021	In a Heartbeat: Prospective Control of Cardiac Responses for Upcoming Action Demands during Biathlon	Ecological Psychology	No	Title abstract or keywords
421		2017	Index to Abstract Descriptors	Psychophysiology	No	Title abstract or keywords
422	F. van Abswoude; N. B. Nuijen; J. van der Kamp; B. Steenbergen	2018	Individual Differences Influencing Immediate Effects of Internal and External Focus Instructions on Children's Motor Performance	Research Quarterly for Exercise & Sport	No	Title abstract or keywords
423	V. V. Wergin; C. J. Mallett; J. Beckmann	2022	Individual vs. Team Sport Failure—Similarities, Differences, and Current Developments	Frontiers in Psychology	No	Title abstract or keywords
424	R. Hayman; R. Polman; E. Borkoles; J. Taylor	2013	The Influence of a Deliberate Practice Intervention on the Putting Performance and Subsequent Practice Behaviours of Aspiring Elite Adolescent Golfers	Talent Development & Excellence	No	Title abstract or keywords

425	R. Cañal-Bruland; J. R. Pijpers; R. D. Oudejans	2010	The influence of anxiety on action-specific perception	Anxiety, Stress & Coping	No	Title abstract or keywords
426	A. Lola; A. Koutsomarkou; G. Tzetzis	2022	Influence of different focus of attention instructions on learning volleyball skills for young novices	Journal of Human Sport & Exercise	No	Title abstract or keywords
427	H. K. C. Faro; D. G. D. S. Machado; H. Bortolotti; P. H. D. do Nascimento; R. C. Moioli; H. M. Elsangedy; E. B. Fontes	2020	Influence of Judo Experience on Neuroelectric Activity During a Selective Attention Task	Frontiers in Psychology	No	Title abstract or keywords
428	M. Weiss; M. Hoegl; M. Gibbert	2013	The influence of material resources on innovation projects: The role of resource elasticity	R and D Management	No	Title abstract or keywords
429	J. Schorer; S. Cobley; D. Büsch; H. Bräutigam; J. Baker	2009	Influences of competition level, gender, player nationality, career stage and playing position on relative age effects	Scandinavian Journal of Medicine & Science in Sports	No	Title abstract or keywords
430	N. Robin; G. R. Coudevylle; O. Hue; L. Toussaint	2018	The influences of tropical climate on imagined walking time	Journal of Cognitive Psychology	No	Title abstract or keywords
431	E. Piveteau; F. Di Rienzo; O. Bolliet; A. Guillot	2023	Inter-task transfer of force gains is facilitated by motor imagery	Frontiers in Neuroscience	No	Title abstract or keywords
432	G. Fontani; S. Migliorini; L. Lodi; E. De Martino; N. Solidakis; F. Corradeschi	2014	Internal-External Motor Imagery and Skilled Motor Actions	Journal of Imagery Research in Sport & Physical Activity	No	Title abstract or keywords
433		2021	International Journal of Sport and Exercise Psychology (IJSEP)	International Journal of Sport & Exercise Psychology	No	Title abstract or keywords
434	N. S. Guest; T. A. VanDusseldorp; M. T. Nelson; J. Grgic; B. J. Schoenfeld; N. D.	2021	International society of sports nutrition position stand: caffeine and exercise performance	Journal of the International Society of Sports Nutrition	No	Title abstract or keywords

	M. Jenkins; S. M. Arent; J. Antonio; J. R. Stout; E. T. Trexler; A. E. Smith-Ryan; E. R. Goldstein; D. S. Kalman; B. I. Campbell					
435	R. B. Kreider; D. S. Kalman; J. Antonio; T. N. Ziegenfuss; R. Wildman; R. Collins; D. G. Candow; S. M. Kleiner; A. L. Almada; H. L. Lopez	2017	International Society of Sports Nutrition position stand: safety and efficacy of creatine supplementation in exercise, sport, and medicine	Journal of the International Society of Sports Nutrition	No	Title abstract or keywords
436	M. S. Boekstein; J. P. Spencer	2013	International trends in health tourism: Implications for thermal spring tourism in the Western Cape Province of South Africa	African Journal for Physical, Health Education, Recreation & Dance	No	Title abstract or keywords
437	T. Devonport; A. Lane; C. L. Fullerton	2016	Introducing Sport Psychology Interventions: Self-Control Implications	Sport Psychologist	No	Title abstract or keywords
438	J. E. Hagan	2021	Investigating pre-competition-related discrete emotions and unaccustomed religious coping among elite student-athletes: Implications for reflexive practice	Religions	No	Title abstract or keywords
439	J. A. Jensen; D. Head; O. Monroe; S. Nestler	2022	Investigating sport league sponsor retention: Results from a semi-parametric hazard model	Sport Management Review	No	Title abstract or keywords
440	C. K. Williams; L. Tremblay; H. Carnahan	2016	It pays to go off-track: Practicing with error-augmenting haptic feedback facilitates learning of a curve-tracing task	Frontiers in Psychology	No	Title abstract or keywords
441	W.-K. Lam; J.-X. Fan; Y. Zheng; W. C.-C. Lee	2019	Joint and plantar loading in table tennis topspin forehand with different footwork	European Journal of Sport Science	No	Title abstract or keywords
442	K. Adler	2003	Keep Your Balance, Inside and Out	Bowling Digest	No	Title abstract or keywords
443	Z. Bańkosz; S. Winiarski	2020	Kinematic Parameters of Topspin Forehand in Table Tennis and Their Inter- and	Journal of Sports	No	Title abstract or keywords

			Intra-Individual Variability	Science & Medicine		
444	J. Finn	2010	Leading Article: Discovering Golf's Innermost Truths: A New Approach to Teaching the Game	International Journal of Sports Science & Coaching	No	Title abstract or keywords
445	Q. Zhang; D. Zhang; P. C. Liao	2022	Leading indicators of mental representation in construction hazard recognition	International Journal of Occupational Safety and Ergonomics	No	Title abstract or keywords
446		2011	Learn To Be A Clutch Putter	Golf Digest	No	Title abstract or keywords
447	M. Wigham; S. Wheatley	2023	Learn, Move, Compete: An alternative approach to mini tennis lessons	Coaching & Sport Science Review	No	Title abstract or keywords
448	C. Porter; D. Greenwood; D. Panchuk; G.-J. Pepping	2020	Learner-adapted practice promotes skill transfer in unskilled adults learning the basketball set shot	European Journal of Sport Science	No	Title abstract or keywords
449	C. J. Wang	2023	Learning and Academic Self-efficacy in Self-regulated Learning: Validation Study with the BOPPPS Model and IRS Methods	Asia-Pacific Education Researcher	No	Title abstract or keywords
450	S. S. Kantak; C. J. Winstein	2012	Learning-performance distinction and memory processes for motor skills: A focused review and perspective	Behavioural Brain Research	No	Title abstract or keywords
451	R. Herrington	2005	Led by captain Kevin Kisner's return to form, consistent Georgia plays to its potential and captures the NCAAs	Golf World	No	Title abstract or keywords
452	B. D. Hickerson; B. A. Beggs	2007	LEISURE TIME BOREDOM: ISSUES CONCERNING COLLEGE STUDENTS	College Student Journal	No	Title abstract or keywords
453	J. Thomas	2018	LESSONS FROM A PLAYER OF THE YEAR SEASON	Golf Magazine	No	Title abstract or keywords
454	A. M. Rymal	2018	Let’s Make It Real: A Commentary on Observation Research	Journal of Motor Learning & Development	No	Title abstract or keywords
455	J. Hawkins	2006	The Lost Generation	Golf World	No	Title abstract or keywords
456	H. R	2005	M.I.A	Golf World	No	Title abstract or keywords
457	A. Shipnuck	2016	MAJOR CATASTROPHE	Sports Illustrated	No	Title abstract or keywords

458	D. J. Spindler; M. S. Allen; S. A. Vella; C. Swann	2017	Manipulating implicit beliefs about decision-making ability affects decision-making performance under submaximal physiological load	Sport, Exercise, and Performance Psychology	No	Title abstract or keywords
459	B. Simpson	2021	Mass Hysteria, Manufacturing Crisis and the Legal Reconstruction of Acceptable Exercise during a Pandemic	Leisure Sciences	No	Title abstract or keywords
460	J. T. Patterson; A. Hart; S. Hansen; M. J. Carter; D. Ditor	2016	Measuring Investment in Learning: Can Electrocardiogram Provide an Indication of Cognitive Effort During Learning?	Perceptual & Motor Skills	No	Title abstract or keywords
461	I. Q. Tay; T. J. Valshtein; N. R. Krott; G. Oettingen	2019	Mental contrasting in DanceSport: The champion's mindset	Psychology of Sport and Exercise	No	Title abstract or keywords
462	M. Khojasteh Moghani; R. Zeidabadi; M. R. Shahabi Kaseb; I. Bahreini Borujeni	2021	Mental Fatigue Reduces the Benefits of Self-Controlled Feedback on Learning a Force Production Task	Perceptual & Motor Skills	No	Title abstract or keywords
463	C. Bédard Thom; F. Guay; C. Trottier	2021	Mental toughness in sport: The Goal-Expectancy-Self-Control (GES) model	Journal of Applied Sport Psychology	No	Title abstract or keywords
464	T. Sitzmann; G. Yeo	2013	A Meta-Analytic Investigation of the Within-Person Self-Efficacy Domain: Is Self-Efficacy a Product of Past Performance or a Driver of Future Performance?	Personnel Psychology	No	Title abstract or keywords
465	M. F. B. Bacelar; J. O. Parma; W. M. Murrah; M. W. Miller	2022	Meta-analyzing enhanced expectancies on motor learning: positive effects but methodological concerns	International Review of Sport and Exercise Psychology	No	Title abstract or keywords
466	Z. Huang; D. H. Choi; B. Lai; Z. Lu; H. Tian	2022	Metaverse-based virtual reality experience and endurance performance in sports economy: Mediating role of mental health and performance anxiety	Frontiers in Public Health	No	Title abstract or keywords
467	E. Bellomo; A. Cooke; G. Gallicchio; C. Ring; J. Hardy	2020	Mind and body: Psychophysiological profiles of instructional and motivational self-talk	Psychophysio logy	No	Title abstract or keywords
468	J. Milton; A. Solodkin; P. Hluštík; S. L. Small	2007	The mind of expert motor performance is cool and focused	NeuroImage	No	Title abstract or keywords
469	K. V. Sparks; M.	2021	Mindfulness, reinvestment, and rowing under	Psychology of	No	Title abstract or

	Kavussanu; R. S. W. Masters; C. Ring		pressure: Evidence for moderated moderation of the anxiety-performance relationship	Sport and Exercise		keywords
470	M. W. Aoyagi; A. B. Cohen; A. Poczwardowski; J. N. Metzler; T. Statler	2018	Models of performance excellence: Four approaches to sport psychology consulting	Journal of Sport Psychology in Action	No	Title abstract or keywords
471	C. Wu; Y.-C. Chen; M. R. Umstattd Meyer	2020	A Moderated Mediation Model of Emotional Labor and Service Performance: Examining the Role of Work–Family Interface and Physically Active Leisure	Human Performance	No	Title abstract or keywords
472	Y. H. Lee; H. Cho	2023	The moderating role of coping strategies in athletic coaches' psychological well-being in response to negative emotions	International Journal of Sports Science & Coaching	No	Title abstract or keywords
473	S. Beattie; C. Dempsey; R. Roberts; T. Woodman; A. Cooke	2017	The moderating role of narcissism on the reciprocal relationship between self-efficacy and performance	Sport, Exercise, and Performance Psychology	No	Title abstract or keywords
474		2008	Monday 21st July 2008	International Journal of Psychology	No	Title abstract or keywords
475		2000	Monday 24th July 2000	International Journal of Psychology	No	Title abstract or keywords
476	L. Rodríguez-Zamora; X. Iglesias; A. Barrero; L. Torres; D. Chaverri; F. A. Rodríguez	2014	Monitoring internal load parameters during competitive synchronized swimming duet routines in elite athletes	Journal of Strength and Conditioning Research	No	Title abstract or keywords
477	Q. Li; X. Wang; S. Wang; Y. Xie; Y. Xie; S. Li	2020	More Flexible Integration of Functional Systems after Musical Training in Young Adults	IEEE Transactions on Neural Systems and Rehabilitation Engineering	No	Title abstract or keywords
478	G. Morone; S. Ghanbari Ghooshchy; C. Pulcini; E.	2022	Motor Imagery and Sport Performance: A Systematic Review on the PETTLEP Model	Applied Sciences (Switzerland)	No	Title abstract or keywords

	Spangu; P. Zoccolotti; M. Martelli; G. F. Spitoni; V. Russo; I. Ciancarelli; S. Paolucci; M. Iosa					
479	M. A. Zurawik	2020	MOVING THROUGH SPACES - LEISURE WALKING AND ITS PSYCHOSOCIAL BENEFITS FOR WELL-BEING: A NARRATIVE REVIEW	Human Movement	No	Title abstract or keywords
480	D. G. Kelty-Stephen; J. Lee; K. R. Cole; R. K. Shields; M. Mangalam	2023	Multifractal Nonlinearity Moderates Feedforward and Feedback Responses to Suprapostural Perturbations	Perceptual & Motor Skills	No	Title abstract or keywords
481	D. G. Kelty-Stephen; E. Lane; L. Bloomfield; M. Mangalam	2023	Multifractal test for nonlinearity of interactions across scales in time series	Behavior Research Methods	No	Title abstract or keywords
482	N. Jacobson; Q. Berleman-Paul; M. Mangalam; D. G. Kelty-Stephen; C. Ralston	2021	Multifractality in postural sway supports quiet eye training in aiming tasks: A study of golf putting	Human Movement Science	No	Title abstract or keywords
483	H. Burianová; L. Marstaller; P. Sowman; G. Tesan; A. N. Rich; M. Williams; G. Savage; B. W. Johnson	2013	Multimodal functional imaging of motor imagery using a novel paradigm	NeuroImage	No	Title abstract or keywords
484	R. Shusterman	2011	MUSCLE MEMORY AND THE SOMAESTUETIC PATHOLOGIES OF EVERYDAY LIFE	Human Movement	No	Title abstract or keywords
485	O. Pickard; P. Burton; H. Yamada; B. Schram; E. F. D. Canetti; R. Orr	2022	Musculoskeletal Disorders Associated with Occupational Driving: A Systematic Review Spanning 2006–2021	International Journal of Environmental Research and Public Health	No	Title abstract or keywords
486	I. T. H. Tso; J. C. L. Law; T. W. L. Wong	2022	Music-Assisted Training for Dart Throwing Novices: Post-Training Effects on Heart Rate and Performance Accuracy	Perceptual & Motor Skills	No	Title abstract or keywords

487		1997	My most memorable race	Runner's World	No	Title abstract or keywords
488	A. Castillo-Rodríguez; C. Muñoz-Arjona; W. Onetti-Onetti	2022	National vs. Non-National Soccer Referee: Physiological, Physical, and Psychological Characteristics	Research Quarterly for Exercise and Sport	No	Title abstract or keywords
489	S. A. L. Spencer; J. L. Van Raalte; B. W. Brewer; M. DeSousa	2022	Negative social exchanges, psychological flexibility, psychological inflexibility, and emotional exhaustion in college and university coaches in the United States	International Journal of Coaching Science	No	Title abstract or keywords
490	S. M. Mousavi; J. Dehghanizade; T. Iwatsuki	2022	Neither Too Easy Nor Too Difficult: Effects of Different Success Criteria on Motor Skill Acquisition in Children	Journal of Sport & Exercise Psychology	No	Title abstract or keywords
491	C. Ruffino; C. Papaxanthis; F. Lebon	2017	Neural plasticity during motor learning with motor imagery practice: Review and perspectives	Neuroscience	No	Title abstract or keywords
492	E. S. Cross; P. J. Schmitt; S. T. Grafton	2007	Neural substrates of contextual interference during motor learning support a model of active preparation	Journal of Cognitive Neuroscience	No	Title abstract or keywords
493	D. Crivelli; M. Balconi	2022	Neuroassessment in Sports: An Integrative Approach for Performance and Potential Evaluation in Athletes	Frontiers in Psychology	No	Title abstract or keywords
494	J. Gold; J. Ciorciari	2021	A neurocognitive model of flow states and the role of cerebellar internal models	Behavioural Brain Research	No	Title abstract or keywords
495	S.-C. Kao; C.-J. Huang; T.-M. Hung	2014	Neurofeedback Training Reduces Frontal Midline Theta and Improves Putting Performance in Expert Golfers	Journal of Applied Sport Psychology	No	Title abstract or keywords
496	C. Collet; F. D. Rienzo; N. Hoyek; A. Guillot	2013	Neurophysiological correlates of motor imagery	Movement and Sports Sciences - Science et Motricite	No	Title abstract or keywords
497	M. Saad; N. A. Husin; S. N. H. Rosman; R. Husain; L. Abdul Latif @ Bapooe	2023	The nexus of community perception on turning idyllic Panchor town into a future river tourism destination	Cogent Social Sciences	No	Title abstract or keywords
498	M. E. d. S. Nunes; U. C. Correa; M. G. T. X. d. Souza; L. Basso; D. B. Coelho; S. Santos	2019	No Improvement on the Learning of Golf Putting By Older Persons With Self-Controlled Knowledge of Performance	Journal of Aging & Physical Activity	No	Title abstract or keywords

499	S. E. Iso-Ahola	2023	Non-consciously processed physical activity for survival versus consciously deliberated exercise for health	Frontiers in Psychology	No	Title abstract or keywords
500		2022	North American Society for the Psychology of Sport and Physical Activity	Journal of Sport & Exercise Psychology	No	Title abstract or keywords
501	R. Meeusen; L. Decroix	2018	Nutritional Supplements and the Brain	International Journal of Sport Nutrition & Exercise Metabolism	No	Title abstract or keywords
502	O. Sollie; K. Holmsen; C. Steinbo; Y. Ommundsen; T. Losnegard	2021	Observational vs coaching feedback on non-dominant whole-body motor skill performance — application to technique training	Scandinavian Journal of Medicine and Science in Sports	No	Title abstract or keywords
503	S. Naaz; S. A. Khan; F. Siddiqui; S. S. Sohail; D. Ø. Madsen; A. Ahmad	2022	OdorTAM: Technology Acceptance Model for Biometric Authentication System Using Human Body Odor	International Journal of Environmental Research and Public Health	No	Title abstract or keywords
504	E. E. Thill; L. Mailhot; J. Mouanda	1998	On how task-contingent rewards, individual differences in causality orientations, and imagery abilities are related to intrinsic motivation and performance	European Journal of Social Psychology	No	Title abstract or keywords
505	Y. L. Dickens; J. V. Raalte; R. T. Hurlburt	2018	On Investigating Self-Talk: A Descriptive Experience Sampling Study of Inner Experience During Golf Performance	Sport Psychologist	No	Title abstract or keywords
506	B. McKay; M. F. B. Bacelar; M. J. Carter	2023	On the Reproducibility of Power Analyses in Motor Behavior Research	Journal of Motor Learning and Development	No	Title abstract or keywords
507	J. G. Milton; S. S. Small; A. Solodkin	2004	On the road to automatic: Dynamic aspects in the development of expertise	Journal of Clinical Neurophysiology	No	Title abstract or keywords
508	A. J. Johnson; T. D. Glover; W. P. Stewart	2009	One Person's Trash is Another Person's Treasure: The Public Place-making of Mount Trashmore""	Journal of Park & Recreation Administration	No	Title abstract or keywords
509	J. Moriarty	2008	One Tough Customer	Golf World	No	Title abstract or

						keywords
510	E. N. Spruit; G. P. H. Band; J. F. Hamming; K. R. Ridderinkhof	2014	Optimal training design for procedural motor skills: A review and application to laparoscopic surgery	Psychological Research	No	Title abstract or keywords
511	Y. Elghoul; F. Bahri; K. Trabelsi; H. Chtourou; M. Frikha; C. C. T. Clark; J. M. Glenn; N. Bragazzi; N. Souissi	2022	Optimizing Motor Learning: Difficulty Manipulation Combined with Feedback-Frequency Enhance Under-Time-Pressure Fine-Motor-Coordination Skill Acquisition and Retention	Journal of Motor Behavior	No	Title abstract or keywords
512	G. Wulf; R. Lewthwaite	2016	Optimizing performance through intrinsic motivation and attention for learning: The OPTIMAL theory of motor learning	Psychonomic Bulletin and Review	No	Title abstract or keywords
513	B. N. Alleoni; A. M. Pellegrini; S. H. Tinos; R. S. Hatore; Q. J. Almeida; C. Mamolo; E. A. Roy; M. S. Jog; D. Ammar; C. P. Gabbard; A. J. Amorose; P. J. K. Smith; D. Anderson-Butcher ; A. Andrade; A. R. Back; D. I. C. Vasconcellos; M. S. Viana; D. P. S. Andrew; J. B. Moore; K. P. Arbour	2005	Oral and Poster	Journal of Sport & Exercise Psychology	No	Title abstract or keywords
514	R. G. Cowden; E. L. Worthington Jr	2019	Overcoming failure in sport: A self-forgiveness framework	Journal of Human Sport & Exercise	No	Title abstract or keywords
515	A. Cohen	2012	The P.A.C.E. Performance Program: Integrating Sport Psychology into Training Programs	Olympic Coach	No	Title abstract or keywords
516	M. Arkush	1996	Pain reliever	Golf World	No	Title abstract or keywords
517		1998	PART V: PSYCHOLOGY	Journal of	No	Title abstract or

				Sports Sciences		keywords
518	D. Jaitner; F. Mess	2019	Participation can make a difference to be competitive in sports: A systematic review on the relation between complex motor development and self-controlled learning settings	International Journal of Sports Science & Coaching	No	Title abstract or keywords
519		2017	Peer-Reviewed Abstracts	Research Quarterly for Exercise & Sport	No	Title abstract or keywords
520	J. C. Norling; J. Sibthorp; E. Ruddell	2008	Perceived Restorativeness for Activities Scale (PRAS): Development and Validation	Journal of Physical Activity & Health	No	Title abstract or keywords
521	M. Weiss; M. Hoegl; M. Gibbert	2014	Perceptions of material resources in innovation projects: What shapes them and how do they matter?	Journal of Product Innovation Management	No	Title abstract or keywords
522	D. P. Broadbent; J. Causer; A. M. Williams; P. R. Ford	2015	Perceptual-cognitive skill training and its transfer to expert performance in the field: Future research directions	European Journal of Sport Science	No	Title abstract or keywords
523	J. Bennett; I. Maynard	2017	Performance blocks in sport: Recommendations for treatment and implications for sport psychology practitioners	Journal of Sport Psychology in Action	No	Title abstract or keywords
524	J. Stead; J. Poolton; D. Alder	2022	Performance slumps in sport: A systematic review	Psychology of Sport and Exercise	No	Title abstract or keywords
525	C. Swann; L. Crust; P. Jackman; S. A. Vella; M. S. Allen; R. Keegan	2017	Performing under pressure: Exploring the psychological state underlying clutch performance in sport	Journal of Sports Sciences	No	Title abstract or keywords
526	R. Wilson; L. Stoner; J. C. Baldi; L. M. Jones	2017	PHYSICAL ACTIVITY NEEDS OF PROSTATE CANCER SURVIVORS COMPARED TO AGE-MATCHED NON-CANCER CONTROLS	New Zealand Journal of Sports Medicine	No	Title abstract or keywords
527	N. J. Robinson; C. Montgomery; L. Swettenham; A. Whitehead	2021	A pilot study investigating cortical haemodynamic and physiological correlates of exercise cognition in trained and untrained cyclists over an incremental self-paced performance test, while thinking aloud	Psychology of Sport and Exercise	No	Title abstract or keywords
528	M. S. Sothmann;	1988	Plasma Catecholamine and Performance	Human	No	Title abstract or

	B. A. Hart; T. S. Horn; A. B. Gustafson		Associations During Psychological Stress: Evidence for Peripheral Noradrenergic Involvement With an Attention-Demanding Task	Performance		keywords
529	H. Pineda	2022	Playing under pressure: EEG monitoring of activation in professional tennis players	Physiology and Behavior	No	Title abstract or keywords
530	P. J. McCarthy	2011	Positive emotion in sport performance: Current status and future directions	International Review of Sport and Exercise Psychology	No	Title abstract or keywords
531		2022	Poster Session 1	Psychophysio logy	No	Title abstract or keywords
532		2013	Poster Session Abstracts	Psychophysio logy	No	Title abstract or keywords
533	J. Porter	2017	PRACTICING WITH GRADUAL INCREASES IN CONTEXTUAL INTERFERENCE: METHODS FOR TESTING THE PREDICTIONS OF THE PARALLEL DEVELOPMENT HYPOTHESIS	Kinesiology	No	Title abstract or keywords
534	R. C. Jackson	2003	Pre-performance routine consistency: temporal analysis of goal kicking in the Rugby Union World Cup	Journal of Sports Sciences	No	Title abstract or keywords
535	K. Kaplanidou; H. Gibson	2010	Predicting Behavioral Intentions of Active Event Sport Tourists: The Case of a Small-scale Recurring Sports Event	Journal of Sport & Tourism	No	Title abstract or keywords
536	N. Sho'ouri	2021	Predicting the success rate of healthy participants in beta neurofeedback: Determining the factors affecting the success rate of individuals	Biomedical Signal Processing and Control	No	Title abstract or keywords
537	A. Boolani; P. J. O'Connor; J. Reid; S. Ma; S. Mondal	2019	Predictors of feelings of energy differ from predictors of fatigue	Fatigue: Biomedicine, Health and Behavior	No	Title abstract or keywords
538	J. M. Wood; D. L. Feltz	2013	Preparatory efficacy effects on practice effort and performance	Open Sports Sciences Journal	No	Title abstract or keywords
539	S. Cotterill	2015	Preparing for Performance: Strategies Adopted Across Performance Domains	Sport Psychologist	No	Title abstract or keywords
540	W. H. van Boom	2011	Price Intransparency, Consumer Decision Making and European Consumer Law	Journal of Consumer Policy	No	Title abstract or keywords
541	G. Wulf; C. H. Shea	2002	Principles derived from the study of simple skills do not generalize to complex skill	Psychonomic Bulletin and	No	Title abstract or keywords

			learning	Review		
542	L. Crocco; P. McCabe; C. Madill	2020	Principles of Motor Learning in Classical Singing Teaching	Journal of Voice	No	Title abstract or keywords
543	E. M. Arroyo-Anlló; J. C. Sánchez; A. R. M. Ventola; P. Ingrand; J. P. Neau; R. Gil	2020	Procedural Learning Improves Cognition in Multiple Sclerosis	Journal of Alzheimer's Disease	No	Title abstract or keywords
544	J. F. Piil; B. Kingma; N. B. Morris; L. Christiansen; L. G. Ioannou; A. D. Flouris; L. Nybo	2021	Proposed framework for forecasting heat-effects on motor-cognitive performance in the Summer Olympics	Temperature	No	Title abstract or keywords
545	J. Penalver-Andres; K. A. Buetler; T. Koenig; R. M. Müri; L. Marchal-Crespo	2021	Providing Task Instructions During Motor Training Enhances Performance and Modulates Attentional Brain Networks	Frontiers in Neuroscience	No	Title abstract or keywords
546	B. C. Berger	1996	Psychological Benefits of an Active Lifestyle: What We Know and What We Need to Know	Quest (00336297)	No	Title abstract or keywords
547	B. Blumenstein; R. Lidor	2008	PSYCHOLOGICAL PREPARATION IN THE OLYMPIC VILLAGE: A FOUR-PHASE APPROACH	International Journal of Sport & Exercise Psychology	No	Title abstract or keywords
548	Y. Tanaka; K. Funase; H. Sekiya; J. Sasaki; Y. M. Tanaka	2014	Psychological pressure facilitates corticospinal excitability: Motor preparation processes and EMG activity in a choice reaction task	International Journal of Sport & Exercise Psychology	No	Title abstract or keywords
549	A. Cooke; M. Kavussanu; D. McIntyre; C. Ring	2010	Psychological, muscular and kinematic factors mediate performance under pressure	Psychophysiology	No	Title abstract or keywords
550	N. M. Hobson; J. Schroeder; J. L. Risen; D. Xygalatas; M. Inzlicht	2018	The Psychology of Rituals: An Integrative Review and Process-Based Framework	Personality and Social Psychology Review	No	Title abstract or keywords
551	P. B. Dos Santos; K. M. Kuczynski; T. do Amaral	2014	Psychophysiological Stress in Under-17 Soccer Players	Journal of Exercise Physiology	No	Title abstract or keywords

	Machado; A. C. Vecchi Osiecki; J. M. Facco Stefanello			Online		
552	B. D. Hatfield; D. M. Landers	1983	Psychophysiology -- A New Direction for Sport Psychology	Journal of Sport Psychology	No	Title abstract or keywords
553	G. L. Read; J. Kim; I. Yen; S. Shuoya; S. Youngji; K. A. Brown	2022	Publicity about Athlete Endorsers Affects Responses to Ads via Motivational Activation	Sport Marketing Quarterly	No	Title abstract or keywords
554	K. A. Shores; S. T. West	2010	Pursuing Leisure During Leisure-Time Physical Activity	Journal of Physical Activity & Health	No	Title abstract or keywords
555	D. M. Wegner; M. Ansfield; D. Pilloff	1998	The putt and the pendulum: Ironic effects of the mental control of action	Psychological Science (0956-7976)	No	Title abstract or keywords
556	P. McDaniel	2000	Rabbit ears	Golf Digest	No	Title abstract or keywords
557	J. Hardy; N. Comoutos; A. Hatzigeorgiadis	2018	Reflections on the Maturing Research Literature of Self-Talk in Sport: Contextualizing the Special Issue	Sport Psychologist	No	Title abstract or keywords
558	S. A. Kay; L. R. Grimm	2017	Regulatory Fit Improves Fitness for People With Low Exercise Experience	Journal of Sport & Exercise Psychology	No	Title abstract or keywords
559	E. Lundkvist; H. Gustafsson; G. Björklund; P. Davis; A. Ivarsson	2021	Relating Competitive Golfers' Perceived Emotions and Performance	Perceptual & Motor Skills	No	Title abstract or keywords
560	C.-H. Lin; F. J. H. Lu; T.-W. Chen; Y. Hsu	2022	Relationship between athlete stress and burnout: a systematic review and meta-analysis	International Journal of Sport & Exercise Psychology	No	Title abstract or keywords
561	K. Berestetska	2020	The Relationship Between Perceived Coaching Behaviors, Intrinsic Motivation, and Scholarship Status on NCAA Division I Tennis Players' Sport Commitment	Journal of Sport Behavior	No	Title abstract or keywords
562	M. Shang-Min; T. Yue; M. Shang-Chun; L.	2012	RELATIONSHIP BETWEEN SEDENTARY AND ACTIVE LEISURE PARTICIPATION AMONG MIDWESTERN COLLEGE	South African Journal for Research in	No	Title abstract or keywords

	Tzi-Li		STUDENTS	Sport, Physical Education & Recreation		
563	M. Gregg; C. Hall	2006	The Relationship of Skill Level and Age to the Use of Imagery by Golfers	Journal of Applied Sport Psychology	No	Title abstract or keywords
564	B. D. Daub; B. D. McLean; A. D. Heishman; A. J. Coutts	2023	The reliability and usefulness of a novel basketball standardized shooting task	International Journal of Sports Science & Coaching	No	Title abstract or keywords
565	T. Kimura; W. Nakano	2019	Repetition of a cognitive task promotes motor learning	Human Movement Science	No	Title abstract or keywords
566		2012	Research Area: Cognitive	International Journal of Psychology	No	Title abstract or keywords
567	I. Popovych; I. Halian; O. Halian; P. Nosov; S. Zinchenko; V. Panok	2021	Research on personality determinants of athletes' mental exhaustion during the ongoing COVID-19 pandemic	Journal of Physical Education & Sport	No	Title abstract or keywords
568	D. E. Aldous	2014	Research opportunities into horticulture and green open space management in the Middle East	Acta Horticulturae	No	Title abstract or keywords
569	N. Konttinen; H. Lyytinen; J. Viitasalo	1998	Rifle-balancing in precision shooting: behavioral aspects of psychophysiological implication	Scandinavian Journal of Medicine & Science in Sports	No	Title abstract or keywords
570	N. M. Hobson; D. Bonk; M. Inzlicht	2017	Rituals decrease the neural response to performance failure	PeerJ	No	Title abstract or keywords
571	L. Uiga; C. M. Capio; R. Donghyun; M. R. Wilson; R. S. W. Masters	2017	THE ROLE OF CONSCIOUSNESS IN BALANCE PERFORMANCE	New Zealand Journal of Sports Medicine	No	Title abstract or keywords
572	M. Wilson; N. C. Smith; P. S. Holmes	2007	The role of effort in influencing the effect of anxiety on performance: Testing the conflicting predictions of processing efficiency theory and the conscious processing hypothesis	British Journal of Psychology	No	Title abstract or keywords
573	M. Wilson; N. Smith; M.	2006	The role of effort in moderating the anxiety – performance relationship: Testing the	Journal of Sports	No	Title abstract or keywords

	Chattington; M. Ford; D. Marple-Horvat		prediction of processing efficiency theory in simulated rally driving	Sciences		
574	W. Wu; D. Yu	2023	The role of individual perceptions in the completion of formalistic tasks	Humanities and Social Sciences Communications	No	Title abstract or keywords
575	R. Kaipa; B. Howard; R. Kaipa; E. Turcat; L. Prema	2020	Role of Massed Versus Distributed Practice in Learning Novel Foreign Language Utterances	Motor Control	No	Title abstract or keywords
576	E. Filho; U. Dobersek; T. A. Husselman	2021	The role of neural efficiency, transient hypofrontality and neural proficiency in optimal performance in self-paced sports: a meta-analytic review	Experimental Brain Research	No	Title abstract or keywords
577	N. Stanger; R. Chettle; J. Whittle; J. Poolton	2018	The Role of Preperformance and In-Game Emotions in Cognitive Interference During Sport Performance: The Moderating Role of Self-Confidence and Reappraisal	Sport Psychologist	No	Title abstract or keywords
578	T. Woodman; P. A. Davis	2008	The Role of Repression in the Incidence of Ironic Errors	Sport Psychologist	No	Title abstract or keywords
579	K. Joon-Ho	2004	The Role of Self-Esteem in Initiating Physical Activity in Consumption Situations	Journal of Sport Management	No	Title abstract or keywords
580	R. Hayman; R. Polman; K. Wharton; E. Borkoles	2020	ROLE STRAIN THEORY: APPLICABILITY IN UNDERSTANDING DEVELOPMENTAL EXPERIENCES OF INTERNATIONAL JUNIOR ACROBATIC GYMNASTS. / TEORIJA DRUŽBENE VLOGE: UPORABNOST IN RAZUMEVANJE RAZVOJA IZKUŠENJ MLADIH AKROBATOV MEDNARODNE KAKOVOSTI	Science of Gymnastics Journal	No	Title abstract or keywords
581	C. H. Shea; G. Wulf	2005	Schema Theory: A Critical Appraisal and Reevaluation	Journal of Motor Behavior	No	Title abstract or keywords
582	S. Mellalieu; G. Trewartha; K. Stokes	2008	Science and rugby union	Journal of Sports Sciences	No	Title abstract or keywords
583	E. Mosley; S. Laborde	2022	A scoping review of heart rate variability in sport and exercise psychology	International Review of Sport and Exercise Psychology	No	Title abstract or keywords

584	R.-N. M. Kassim; M. A. Asbullah; M. H. Basal; M. N. Ab Aziz; H. C. Mat; N. S. Nawai; B. S. Zaman	2022	Scrutinizing the Behaviour and Commitment of a Mixed Martial Art Fighting Online Gamer. / Ocena zachowań i zaangażowania graczy uprawiających elektroniczne mieszane sztuki walki	Ido Movement for Culture. Journal of Martial Arts Anthropology	No	Title abstract or keywords
585	T. Glenn	2020	Security in a safety pin	Families, Systems and Health	No	Title abstract or keywords
586	A. Ede; P. J. Sullivan; D. L. Feltz	2017	Self-doubt: Uncertainty as a motivating factor on effort in an exercise endurance task	Psychology of Sport and Exercise	No	Title abstract or keywords
587	J. T. Fairbrother; P. G. Post; S. J. Whalen	2016	Self-reported responses to player profile questions show consistency with the use of complex attentional strategies by expert horseshoe pitchers	Frontiers in Psychology	No	Title abstract or keywords
588	E. D. Van Dyke; J. L. Van Raalte; E. M. Mullin; B. W. Brewer	2018	Self-talk and competitive balance beam performance	Sport Psychologist	No	Title abstract or keywords
589	A. Hatzigeorgiadis; N. Zourbanos; E. Galanis; Y. Theodorakis	2011	Self-talk and sports performance: A meta-analysis	Perspectives on Psychological Science	No	Title abstract or keywords
590	M. J. Mitten	2014	SEVENTH CIRCUIT AND WISCONSIN SPORTS LAW JURISPRUDENCE	Marquette Sports Law Review	No	Title abstract or keywords
591	R. Sides; G. Chow; G. Tenenbaum	2017	Shifts in Adaptation: The Effects of Self-Efficacy and Task Difficulty Perception	Journal of Clinical Sport Psychology	No	Title abstract or keywords
592	K. Bianchi; M. Brillinger; J. T. Patterson	2022	Size Perception of a Sport Target as a Function of Practice Success Conditions	Frontiers in Psychology	No	Title abstract or keywords
593	C. Ferguson; H. J. Carson; D. Collins	2023	Skill execution errors: an “it depends” perspective on their role, type and use when coaching for player development in sport	Sports Coaching Review	No	Title abstract or keywords
594	X. Zhou; M. M. P. Rana	2012	Social benefits of urban green space: A conceptual framework of valuation and accessibility measurements	Management of Environmenta l Quality	No	Title abstract or keywords
595	M. Silver	1997	Sound and fury	Sports Illustrated	No	Title abstract or keywords
596	N. Schaffert; B. Oldag; P. Cesari	2020	Sound matters: The impact of auditory deprivation on movement precision in rowing	European Journal of	No	Title abstract or keywords

				Sport Science		
597	M. Ćosić; V. Koprivica	2010	SPECIFIČNOSTI STRUKTURE TRENINGA KOD SPORTISTA SA OŠTEĆENIM VIDOM	Proceedings of the Faculty of Physical Education, University of Banja Luka	No	Title abstract or keywords
598		2013	Sport and Exercise Psychology	Journal of Sport & Exercise Psychology	No	Title abstract or keywords
599	D. P. Schary; C. Lundqvist	2021	SPORT PARTICIPATION'S EFFECT ON ATHLETE MENTAL HEALTH DURING COVID-19 RESTRICTIONS	Journal of Contemporary Athletics	No	Title abstract or keywords
600	S. Ionta; A. Ferretti; A. Merla; A. Tartaro; G. L. Romani	2010	Step-by-step: The effects of physical practice on the neural correlates of locomotion imagery revealed by fMRI	Human Brain Mapping	No	Title abstract or keywords
601	S. Jones	2023	The storm before the calm	Golfdom	No	Title abstract or keywords
602	E. Galanis; A. Hatzigeorgiadis; F. Charachousi; A. T. Latinjak; N. Comoutos; Y. Theodorakis	2022	Strategic Self-Talk Assists Basketball Free Throw Performance Under Conditions of Physical Exertion	Frontiers in Sports and Active Living	No	Title abstract or keywords
603	N. V. Weston; R. Thelwell; S. Bond; N. Hutchings	2009	Stress and Coping in Single-Handed Round-the-World Ocean Sailing	Journal of Applied Sport Psychology	No	Title abstract or keywords
604	P. Olusoga; J. Butt; I. Maynard; K. Hays	2010	Stress and Coping: A Study of World Class Coaches	Journal of Applied Sport Psychology	No	Title abstract or keywords
605	M. A. Thompson; J. Toner; J. L. Perry; R. Burke; A. R. Nicholls	2020	Stress appraisals influence athletic performance and psychophysiological response during 16.1 km cycling time trials	Psychology of Sport and Exercise	No	Title abstract or keywords
606	A. Watts	1987	STRESS IN RETIREMENT AND ITS MANAGEMENT	Stress Medicine	No	Title abstract or keywords
607	P. M. Young; S. Partington; M. A. Wetherell; A. St Clair Gibson; E. Partington	2014	Stressors and Coping Strategies of UK Firefighters during On-duty Incidents	Stress & Health: Journal of the International Society for the	No	Title abstract or keywords

				Investigation of Stress		
608	J. Stynes; M. Pink; E. Aumand	2017	Stressors, coping strategies and effectiveness: A study of Special Olympics coaches at a major sporting event	International Sports Studies	No	Title abstract or keywords
609	Y. Yeo Kyung	2019	A Study on the Korean equestrian participation process based on revised Psychological Continuum Model	International Journal of Applied Sports Sciences	No	Title abstract or keywords
610		2001	Summary of Symposium: Attention, Skill Development, and Brainwave Changes During Sports Training	Journal of Sport & Exercise Psychology	No	Title abstract or keywords
611	T. Rosaforte	2005	Summer solutions	Golf World	No	Title abstract or keywords
612	S. D. Ringenbach; B. D. Ulrich; R. M. Angulo-Barroso; D. A. Ulrich; N. Virji-Babul; B. K. V. Maraj; M. Brown; A. Richards; S. Slipp; T. Horn; A. L. Smith; L. Williams; S. Glenn; S. Ullrich-French; E. G. Walker Ii; K. S. Hurley; W. Campbell; D. Burton; A. Cox	2003	SUPPLEMENT TO JOURNAL OF SPORT & EXERCISE PSYCHOLOGY	Journal of Sport & Exercise Psychology	No	Title abstract or keywords
613	S. A. Vella; R. E. Braithewaite; L. A. Gardner; C. M. Spray	2016	A systematic review and meta-analysis of implicit theory research in sport, physical activity, and physical education	International Review of Sport and Exercise Psychology	No	Title abstract or keywords
614	K. L. Payne; M. R. Wilson; S. J. Vine	2019	A systematic review of the anxiety-attention relationship in far-aiming skills	International Review of Sport and Exercise Psychology	No	Title abstract or keywords
615	S. Schaefer; D.	2020	Table Tennis Experts Outperform Novices in a	Journal of	No	Title abstract or

	Scornaienchi		Demanding Cognitive-Motor Dual-Task Situation	Motor Behavior		keywords
616	N. T. Ong; J. Hawke; N. J. Hodges	2019	Target size manipulations affect error-processing duration and success perceptions but not behavioural indices of learning	Brain Sciences	No	Title abstract or keywords
617	K. Saxe; L. Beasley; R. Abdulhussein	2022	Team Environments Influence Student-Athlete Mental Health Through Mesolevel Interactions: An Ecological Systems Perspective	Journal of Issues in Intercollegiate Athletics	No	Title abstract or keywords
618	J. Fritsch; A. Hatzigeorgiadis; D. Jekauc; A. T. Latinjak	2020	A Theoretical Article on Self-Talk in Sports Psychology	Zeitschrift fur Sportpsychologie	No	Title abstract or keywords
619	R. D. Oudejans; W. Kuijpers; C. Kooijman; F. Bakker	2011	Thoughts and attention of athletes under pressure: skill-focus or performance worries?	Anxiety, Stress & Coping	No	Title abstract or keywords
620	T. Rosaforte; J. Strege	2003	Tired and true	Golf World	No	Title abstract or keywords
621	Y. K. Lee; C. K. Lee; J. Choi; S. M. Yoon; R. J. Hart	2014	Tourism's role in urban regeneration: Examining the impact of environmental cues on emotion, satisfaction, loyalty, and support for Seoul's revitalized Cheonggyecheon stream district	Journal of Sustainable Tourism	No	Title abstract or keywords
622	T. Kim; A. Duhachek; K. Herd; S. Kim	2022	Toward a goal-based paradigm of contagion	European Journal of Marketing	No	Title abstract or keywords
623	D. Jekauc; J. Fritsch; A. T. Latinjak	2021	Toward a Theory of Emotions in Competitive Sports	Frontiers in Psychology	No	Title abstract or keywords
624	K. A. Ericsson	2020	Towards a science of the acquisition of expert performance in sports: Clarifying the differences between deliberate practice and other types of practice	Journal of Sports Sciences	No	Title abstract or keywords
625	F. Malouin; P. L. Jackson; C. L. Richards	2013	Towards the integration of mental practice in rehabilitation programs. A critical review	Frontiers in Human Neuroscience	No	Title abstract or keywords
626	D. Longman; J. T. Stock; J. C. K. Wells	2017	A trade-off between cognitive and physical performance, with relative preservation of brain function	Scientific Reports	No	Title abstract or keywords
627	S. Perrey	2022	Training Monitoring in Sports: It Is Time to Embrace Cognitive Demand	Sports (2075-4663)	No	Title abstract or keywords
628	L. Resnik; S. L. Klinger; K. Korp;	2014	TRAINING PROTOCOL FOR POWERED SHOULDER PROSTHESIS	Journal of Rehabilitation	No	Title abstract or keywords

	L. S. Walters			Research & Development		
629	C. Degen; J. Schröder	2014	Training-induced cerebral changes in the elderly	Restorative Neurology and Neuroscience	No	Title abstract or keywords
630	S. Ryan	2023	The Trials of TURNING PRO	NCGA Golf	No	Title abstract or keywords
631	D. G. Kelty-Stephen; M. Mangalam	2022	Turing's cascade instability supports the coordination of the mind, brain, and behavior	Neuroscience and Biobehavioral Reviews	No	Title abstract or keywords
632	Z. Avner; P. Markula; J. Denison	2017	Understanding Effective Coaching: A Foucauldian Reading of Current Coach Education Frameworks	International Sport Coaching Journal	No	Title abstract or keywords
633	G. Zan; A. M. Lee; L. Harrison Jr	2008	Understanding Students' Motivation in Sport and Physical Education: From the Expectancy-Value Model and Self-Efficacy Theory Perspectives	Quest (00336297)	No	Title abstract or keywords
634	B. H. Cheng; J. M. McCarthy	2018	Understanding the dark and bright sides of anxiety: A theory of workplace anxiety	Journal of Applied Psychology	No	Title abstract or keywords
635	C. Mackintosh; G. Griggs; R. Tate	2018	Understanding the growth in outdoor recreation participation: an opportunity for sport development in the United Kingdom	Managing Sport & Leisure	No	Title abstract or keywords
636	A. Piras; M. Timmis; A. Trofè; M. Raffi	2021	Understanding the underlying mechanisms of Quiet Eye: The role of microsaccades, small saccades and pupil-size before final movement initiation in a soccer penalty kick	European Journal of Sport Science	No	Title abstract or keywords
637	K. Rocha; V. Marinho; F. Magalhães; V. Carvalho; T. Fernandes; M. Ayres; E. Crespo; B. Velasques; P. Ribeiro; M. Cagy; V. H. Bastos; D. S. Gupta; S. Teixeira	2020	Unskilled shooters improve both accuracy and grouping shot having as reference skilled shooters cortical area: An EEG and tDCS study	Physiology and Behavior	No	Title abstract or keywords
638	P. Gaudreau; A. Nicholls; A. R. Levy	2010	The Ups and Downs of Coping and Sport Achievement: An Episodic Process Analysis of Within-Person Associations	Journal of Sport & Exercise Psychology	No	Title abstract or keywords

639	T. Gabbett; M. Wake; B. Abernethy	2011	Use of dual-task methodology for skill assessment and development: Examples from rugby league	Journal of Sports Sciences	No	Title abstract or keywords
640	Ł. Rydzik; W. Wąsacz; T. Ambroży; N. Javdaneh; K. Brydak; M. Kopańska	2023	The Use of Neurofeedback in Sports Training: Systematic Review	Brain Sciences	No	Title abstract or keywords
641	D. Yun; L. Zhang; Y. Qiu; R. Schinke; J. Liu	2023	The usefulness of the useless: How ritualized behavior improves self-control under competition pressure	Journal of Applied Sport Psychology	No	Title abstract or keywords
642	M. Noetel; J. Ciarrochi; B. Sahdra; C. Lonsdale	2019	Using genetic algorithms to abbreviate the Mindfulness Inventory for Sport: A substantive-methodological synthesis	Psychology of Sport and Exercise	No	Title abstract or keywords
643	S. L. Bell; C. Phoenix; R. Lovell; B. W. Wheeler	2015	Using GPS and geo-narratives: a methodological approach for understanding and situating everyday green space encounters	Area	No	Title abstract or keywords
644	C. J. Wakefield; D. Smith; E. Hogard; R. Ellis; C. Parry	2020	Using PETTLEP imagery as a simulation technique in nursing: Research and guidelines	Nurse Education in Practice	No	Title abstract or keywords
645	D. Wright; P. Holmes; D. Smith	2011	Using the Movement-Related Cortical Potential to Study Motor Skill Learning	Journal of Motor Behavior	No	Title abstract or keywords
646	R. Hayman; R. Polman; J. Taylor	2012	The validity of retrospective recall in assessing practice regimes in golf	International Journal of Sport & Exercise Psychology	No	Title abstract or keywords
647	B. G. C. Dellaert; K. Lindberg	2003	Variations in Tourist Price Sensitivity: A Stated Preference Model to Capture the Joint Impact of Differences in Systematic Utility and Response Consistency	Leisure Sciences	No	Title abstract or keywords
648	S. Schaper; L. v. d. Kaaden; V. d. Boode; G. Savelsbergh	2020	Visual gaze behaviour during free-kicks in football	International Journal of Sports Science & Coaching	No	Title abstract or keywords
649	T. Hülsdünker; A. Mierau	2021	Visual Perception and Visuomotor Reaction Speed Are Independent of the Individual Alpha Frequency	Frontiers in Neuroscience	No	Title abstract or keywords
650	A. Piras; M. A.	2021	Visual Strategies Underpinning the	Journal of	No	Title abstract or

	Timmis; A. Trofè; M. Raffi		Spatiotemporal Demands During Visuomotor Tasks in Predicting Ball Direction	Sport & Exercise Psychology		keywords
651		2012	Waggle Room	Golf World	No	Title abstract or keywords
652	S. Liang; B. Lan; R. Li; M. Zhang; Y. Chu; L. Teng; L. He	2023	Waking up "We" or "I"? How Start Temporal Landmarks Influence Arousal Product Preferences	Behavioral Sciences	No	Title abstract or keywords
653	A. Baca; B. Schwartz	2016	Wearables and Apps -- Modern Diagnostic Frameworks for Health Promotion through Sport. / Wearables und Apps als moderne diagnostische Frameworks zur Gesundheitsförderung durch Sport	German Journal of Sports Medicine / Deutsche Zeitschrift fur Sportmedizin	No	Title abstract or keywords
654	H. C. Wu; C. F. Wei; L. Y. Tseng; C. C. Cheng	2018	What drives green brand switching behavior?	Marketing Intelligence and Planning	No	Title abstract or keywords
655	E. A. Sanli; T. D. Lee	2014	What Roles Do Errors Serve in Motor Skill Learning? An Examination of Two Theoretical Predictions	Journal of Motor Behavior	No	Title abstract or keywords
656	J. Hawkins	2006	What Should Phil do next?	Golf World	No	Title abstract or keywords
657	Y. Kluch	2023	Why don't more college athletes engage in activism? A multilevel analysis of barriers to activism in the hegemonic arena of intercollegiate sport	Sport Management Review	No	Title abstract or keywords
658	N. W. Van Yperen	2009	Why Some Make It and Others Do Not: Identifying Psychological Factors That Predict Career Success in Professional Adult Soccer	Sport Psychologist	No	Title abstract or keywords
659	M. Söderberg	2014	Willingness to Pay for Nontraditional Attributes Among Participants of a Long-Distance Running Race	Journal of Sports Economics	No	Title abstract or keywords
660	A. García-Mas; E. Ortega; J. Ponseti; C. de Teresa; D. Cárdenas	2016	Workload and cortisol levels in helicopter combat pilots during simulated flights. / Carga mental y niveles de cortisol en pilotos de helicóptero de combate en vuelos simulados	Revista Andaluza de Medicina del Deporte	No	Title abstract or keywords
661	J. Woolf; J. C. Dixon	2017	You're Hired! A Hiring Simulation for Sport Management Students That Incorporates the Hidden Profile Phenomenon	Sport Management Education Journal (Human Kinetics)	No	Title abstract or keywords
662	D. Gould; S.	2011	Young Athletes Perceptions of the	International	No	Title abstract or

	Carson		Relationship between Coaching Behaviors and Developmental Experiences	Journal of Coaching Science		keywords
663	A.-F. StĂNcioiu; N. Teodorescu; I. O. N. PÂRgaru; A. Botos; C.-A. BĂLtescu	2013	Young people's motivations and preferences for sports tourism	Journal of Physical Education & Sport	No	Title abstract or keywords
664	M. F. N. Smith, Alex J.; Baker, Mistrelle R.	2012	Effect of Acute Mild Dehydration on Cognitive-Motor Performance in Golf		No	Without any form of intervention
665	A. Oliver; P. J. McCarthy; L. Burns	2020	A Grounded-Theory Study of Meta-Attention in Golfers	SPORT PSYCHOLOGIST	No	Without any form of intervention
666	A. G. Wood; M. J. Turner; J. B. Barker; S. J. Higgins	2017	Investigating the effects of irrational and rational self-statements on motor-skill and hazard-perception performance	Sport, Exercise, and Performance Psychology	No	Without any form of intervention
667	H. J. Carson; D. Collins	2015	Tracking Technical Refinement in Elite Performers: The Good, the Better, and the Ugly	International Journal of Golf Science	No	Without any form of intervention
668	Wang Xi;Zhang Shaoping;Chen Chong;Gu Yi;	2019	Analysis of Application Prospect of Aromatic Plants in Golf Courses	Journal of Green Science and Technology	No	Without any form of intervention
669	H. J. Carson; D. Collins; J. Richards	2016	Initiating technical refinements in high-level golfers: Evidence for contradictory procedures	Eur J Sport Sci	No	Without any form of intervention
670	H. J. Carson; D. Collins; J. Richards	2014	Intra-individual movement variability during skill transitions: A useful marker?	European Journal of Sport Science	No	Without any form of intervention
671	S. L. Beilock; J. A. Afremow; A. L. Rabe; T. H. Carr	2001	Don't miss!" - The debilitating effects of suppressive imagery on golf putting performance"	JOURNAL OF SPORT & EXERCISE PSYCHOLOGY	No	Without any form of intervention
672	C. Swann; R. Keegan; L. Crust; D. Piggott	2016	Psychological states underlying excellent performance in professional golfers: “Letting it happen” vs. “making it happen”	Psychology of Sport and Exercise	No	Without any form of intervention
673	R. M. Walters-Symons; M. R. Wilson; S. J. Vine	2017	The quiet eye supports error recovery in golf putting	Psychology of Sport and Exercise	No	Without any form of intervention
674	N. N. Toskovic	2001	Alterations in selected measures of mood with a single bout of dynamic Taekwondo exercise	Perceptual and Motor	No	Conclusions are not related to the

			in college-age students	Skills		level of mental fatigue or any form of golf performance
675	B. S. DeCouto; A. M. Williams; K. R. Lohse; S. H. Creem-Regehr; D. L. Strayer; P. C. Fino	2021	Anxiety does not always affect balance: the predominating role of cognitive engagement in a video gaming task	Experimental Brain Research	No	Conclusions are not related to the level of mental fatigue or any form of golf performance
676	W. G. Hopkins	2021	Athlete Monitoring and Much More at the Virtual 26th Annual Meeting of the European College of Sport Science	Sportscience	No	Conclusions are not related to the level of mental fatigue or any form of golf performance
677	R. Mullen; A. Faull; E. S. Jones; K. Kingston	2012	Attentional focus and performance anxiety: Effects on simulated race-driving performance and heart rate variability	Frontiers in Psychology	No	Conclusions are not related to the level of mental fatigue or any form of golf performance
678	M. Mikicin; M. Kowalczyk	2015	Audio-Visual and Autogenic Relaxation Alter Amplitude of Alpha EEG Band, Causing Improvements in Mental Work Performance in Athletes	APPLIED PSYCHOPH YSIOLOGY AND BIOFEEDBA CK	No	Conclusions are not related to the level of mental fatigue or any form of golf performance
679	T. D. Lee; S. P. Swinnen; D. J. Serrien	1994	Cognitive effort and motor learning	Quest	No	Conclusions are not related to the level of mental fatigue or any form of golf performance
680	M. Wilson; M. Chattington; D. E. Marple-Horvat; N. C. Smith	2007	A comparison of self-focus versus attentional explanations of choking	Journal of Sport & Exercise Psychology	No	Conclusions are not related to the level of mental fatigue or any form of golf performance
681	S. E. Iso-ahola	2015	Conscious versus Nonconscious Mind and Leisure	Leisure Sciences	No	Conclusions are not related to the level of mental fatigue or any form of golf

						performance
682	J. Gregersen; A. Hatzigeorgiadis; E. Galanis; N. Comoutos; A. Papaioannou	2017	Countering the consequences of ego depletion: The effects of self-talk on selective attention	Journal of Sport and Exercise Psychology	No	Conclusions are not related to the level of mental fatigue or any form of golf performance
683	R. Boat; C. Sunderland; S. B. Cooper	2021	Detrimental effects of prior self-control exertion on subsequent sporting skill performance	Scandinavian Journal of Medicine and Science in Sports	No	Conclusions are not related to the level of mental fatigue or any form of golf performance
684	N. J. Hodges; K. R. Lohse	2020	Difficulty is a Real Challenge: A Perspective on the Role of Cognitive Effort in Motor Skill Learning	Journal of Applied Research in Memory and Cognition	No	Conclusions are not related to the level of mental fatigue or any form of golf performance
685	N. Sho'ouri; M. Firoozabadi; K. Badie	2020	The effect of beta/alpha neurofeedback training on imitating brain activity patterns in visual artists	Biomedical Signal Processing and Control	No	Conclusions are not related to the level of mental fatigue or any form of golf performance
686	L. Filipas; F. Mottola; G. Tagliabue; A. La Torre	2018	The effect of mentally demanding cognitive tasks on rowing performance in young athletes	Psychology of Sport and Exercise	No	Conclusions are not related to the level of mental fatigue or any form of golf performance
687	L. Giessing; M. O. Frenkel; C. Zinner; J. Rummel; A. Nieuwenhuys; C. Kasperk; M. Brune; F. A. Engel; H. Plessner	2019	Effects of coping-related traits and psychophysiological stress responses on police recruits' shooting behavior in reality-based scenarios	Frontiers in Psychology	No	Conclusions are not related to the level of mental fatigue or any form of golf performance
688	M. C. J. Hoskens; L. Uiga; A. Cooke; C. M. Capio; R. S. W. Masters	2022	The Effects of Fatigued Working Memory Functions on Hypothesis Testing During Acquisition of a Motor Skill	Journal of Experimental Psychology: General	No	Conclusions are not related to the level of mental fatigue or any form of golf performance

689	C. Englert; K. Zwemmer; A. Bertrams; R. R. D. Oudejans	2015	Ego Depletion and Attention Regulation Under Pressure: Is a Temporary Loss of Self-Control Strength Indeed Related to Impaired Attention Regulation?	Journal of Sport & Exercise Psychology	No	Conclusions are not related to the level of mental fatigue or any form of golf performance
690	C. Hertzog; A. F. Kramer; R. S. Wilson; U. Lindenberger	2008	Enrichment effects on adult cognitive development: Can the functional capacity of older adults be preserved and enhanced?	Psychological Science in the Public Interest, Supplement	No	Conclusions are not related to the level of mental fatigue or any form of golf performance
691	H. Ramezanzade; E. Saemi; D. P. Broadbent; J. M. Porter	2022	An Examination of the Contextual Interference Effect and the Errorless Learning Model during Motor Learning	Journal of Motor Behavior	No	Conclusions are not related to the level of mental fatigue or any form of golf performance
692	M. S. Rosenbaum; C. Massiah	2011	An expanded servicescape perspective	Journal of Service Management	No	Conclusions are not related to the level of mental fatigue or any form of golf performance
693	A. Khacharem; B. Zoudji; S. Kalyuga; H. Ripoll	2013	The Expertise Reversal Effect for Sequential Presentation in Dynamic Soccer Visualizations	Journal of Sport & Exercise Psychology	No	Conclusions are not related to the level of mental fatigue or any form of golf performance
694	A. Boolani; M. Manierre	2019	An exploratory multivariate study examining correlates of trait mental and physical fatigue and energy	Fatigue: Biomedicine, Health and Behavior	No	Conclusions are not related to the level of mental fatigue or any form of golf performance
695	T. Gabbett; D. Jenkins; B. Abernethy	2009	Game-Based Training for Improving Skill and Physical Fitness in Team Sport Athletes	International Journal of Sports Science & Coaching	No	Conclusions are not related to the level of mental fatigue or any form of golf performance
696	D. Veličković; D. Radovanović	2018	GENDER DIFFERENCES IN CHESS PERFORMANCE	Facta Universitatis: Series	No	Conclusions are not related to the level of mental

				Physical Education & Sport		fatigue or any form of golf performance
697	M. A. Rendell; R. S. W. Masters; D. Farrow; T. Morris	2011	An implicit basis for the retention benefits of random practice	Journal of Motor Behavior	No	Conclusions are not related to the level of mental fatigue or any form of golf performance
698	J. D. Graham; M. W. L. Sonne; S. R. Bray	2014	It wears me out just imagining it! Mental imagery leads to muscle fatigue and diminished performance of isometric exercise	Biological Psychology	No	Conclusions are not related to the level of mental fatigue or any form of golf performance
699	A. A. Mohd-Any; H. Winklhofer; C. Ennew	2015	Measuring Users' Value Experience on a Travel Website (e-Value): What Value Is Cocreated by the User?	Journal of Travel Research	No	Conclusions are not related to the level of mental fatigue or any form of golf performance
700	B. Thürer; C. Stockinger; F. Putze; T. Schultz; T. Stein	2017	Mechanisms within the parietal cortex correlate with the benefits of random practice in motor adaptation	Frontiers in Human Neuroscience	No	Conclusions are not related to the level of mental fatigue or any form of golf performance
701	H. Herrebrøden; T. Espeseth; L. Bishop	2023	Mental Effort in Elite and Nonelite Rowers	Journal of Sport and Exercise Psychology	No	Conclusions are not related to the level of mental fatigue or any form of golf performance
702	C. Jiang; V. K. Ranganathan; J. Zhang; V. Siemionow; G. H. Yue	2016	Motor effort training with low exercise intensity improves muscle strength and descending command in aging	Medicine (United States)	No	Conclusions are not related to the level of mental fatigue or any form of golf performance
703	L. Bishop; A. R. Jensenius; B. Laeng	2021	Musical and Bodily Predictors of Mental Effort in String Quartet Music: An Ecological Pupillometry Study of Performers and Listeners	Frontiers in Psychology	No	Conclusions are not related to the level of mental fatigue or any form of golf performance

704	K. B. Knox; L. Clay; K. Stuart-Kobitz; D. Nickel	2020	Perspectives on walking from people with multiple sclerosis and reactions to video self-observation	Disability and Rehabilitation	No	Conclusions are not related to the level of mental fatigue or any form of golf performance
705	C. Swann; M. J. Schweickle; G. E. Peoples; S. G. Goddard; C. Stevens; S. A. Vella	2022	The potential benefits of nonspecific goals in physical activity promotion: Comparing open, do-your-best, and as-well-as-possible goals in a walking task	Journal of Applied Sport Psychology	No	Conclusions are not related to the level of mental fatigue or any form of golf performance
706	Z. Khodakarami; M. Firoozabadi	2020	Psychological, neurophysiological, and mental factors associated with gamma-enhancing neurofeedback success	Basic and Clinical Neuroscience	No	Conclusions are not related to the level of mental fatigue or any form of golf performance
707	R. J. Apparies; T. C. Riniolo; S. W. Porges	1998	A psychophysiological investigation of the effects of driving longer-combination vehicles	Ergonomics	No	Conclusions are not related to the level of mental fatigue or any form of golf performance
708	A. Hatzigeorgiadis; E. Galanis	2017	Self-talk effectiveness and attention	Current Opinion in Psychology	No	Conclusions are not related to the level of mental fatigue or any form of golf performance
709	M. Anderson; K. M. Petit; A. C. Bretzin; R. J. Elbin; K. L. Stephenson; T. Covassin	2020	Sport Concussion Assessment Tool Symptom Inventory: Healthy and Acute Postconcussion Symptom Factor Structures	Journal of Athletic Training (Allen Press)	No	Conclusions are not related to the level of mental fatigue or any form of golf performance
710	A. Moran; A. Quinn; M. Campbell; B. Rooney; N. Brady; C. Burke	2016	Using pupillometry to evaluate attentional effort in quiet eye: A preliminary investigation	Sport, Exercise, and Performance Psychology	No	Conclusions are not related to the level of mental fatigue or any form of golf performance
711	J. V. V. Parr; S. J. Vine; M. R. Wilson; N. R.	2019	Visual attention, EEG alpha power and T7-Fz connectivity are implicated in prosthetic hand control and can be optimized through gaze	Journal of NeuroEngineering and	No	Conclusions are not related to the level of mental

	Harrison; G. Wood		training	Rehabilitation		fatigue or any form of golf performance
712	C. N. Macrae; B. M. Christian; M. Golubickis; M. Karanasiou; L. Troksiarova; D. L. McNamara; L. K. Miles	2014	When do i wear me out? Mental simulation and the diminution of self-control	Journal of Experimental Psychology: General	No	Conclusions are not related to the level of mental fatigue or any form of golf performance
713	H. Scholten	2023	You've Got Three Choices: Give in, Give up, or Give it All You've Got: Does Contest Heterogeneity Affect Effort in Individual Competitions?	Journal of Sports Economics	No	Conclusions are not related to the level of mental fatigue or any form of golf performance
714	J. Habay; J. Van Cutsem; J. Verschueren; S. De Bock; M. Proost; J. De Wachter; B. Tassignon; R. Meeusen; B. Roelands	2021	Mental Fatigue and Sport-Specific Psychomotor Performance: A Systematic Review	Sports Medicine	No	Review Article
715	T. van Duijn; A. Cooke; E. Bellomo; R. Masters	2017	EEG COHERENCE AND CONSCIOUS MOTOR PROCESSING IN GOLF PUTTING BEGINNERS	New Zealand Journal of Sports Medicine	No	No full text
716	E. Carnegie; D. Marchant; S. Towers; P. Ellison	2020	Beyond visual fixations and gaze behaviour. Using pupillometry to examine the mechanisms in the planning and motor performance of a golf putt	Hum Mov Sci	Yes	
717	W. K. Lam; R. S. Masters; J. P. Maxwell	2010	Cognitive demands of error processing associated with preparation and execution of a motor skill	Conscious Cogn	Yes	
718	O. R. Runswick; M. Jewiss; B. Sharpe; J. S. North	2021	Context Affects Quiet Eye Duration and Motor Performance Independent of Cognitive Effort	JOURNAL OF SPORT & EXERCISE PSYCHOLOGY	Yes	
719	E. J. Stevenson; P. R. Hayes; S. J. Allison	2009	The effect of a carbohydrate-caffeine sports drink on simulated golf performance	Applied Physiology, Nutrition & Metabolism	Yes	

720	P. W. Mumford; A. C. Tribby; C. N. Poole; V. J. Dalbo; A. T. Scanlan; J. R. Moon; M. D. Roberts; K. C. Young	2016	Effect of Caffeine on Golf Performance and Fatigue during a Competitive Tournament	Med Sci Sports Exerc	Yes	
721	E. Galanis; L. Nurkse; J. Kooijman; E. Papagiannis; A. Karathanasi; N. Comoutos; Y. Theodorakis; A. Hatzigeorgiadis	2022	Effects of a Strategic Self-Talk Intervention on Attention Functions and Performance in a Golf Task under Conditions of Ego Depletion	SUSTAINABILITY	Yes	
722	Y. Nagashima; K. Ehara; Y. Ehara; A. Mitsume; K. Kubo; S. Mineo	2023	Effects of Continuous Carbohydrate Intake with Gummies during the Golf Round on Interstitial Glucose, Golf Performance, and Cognitive Performance of Competitive Golfers: A Randomized Repeated-Measures Crossover Design	Nutrients	Yes	
723	M. Shin; Y. Kim; S. Park	2019	Effects of State Anxiety and Ego Depletion on Performance Change in Golf Putting: A Hierarchical Linear Model Application	Perceptual & Motor Skills	Yes	
724	M. J. Campbell; A. P. Moran; N. Bargary; S. Surmon; L. Bressan; I. C. Kenny	2019	Pupillometry During Golf Putting: A New Window on the Cognitive Mechanisms Underlying Quiet Eye	SPORT EXERCISE AND PERFORMANCE PSYCHOLOGY	Yes	
725	B. K. Doan; R. U. Newton; W. J. Kraemer; Y. H. Kwon; T. P. Scheet	2007	Salivary cortisol, testosterone, and T/C ratio responses during a 36-hole golf competition	Int J Sports Med	Yes	

Literature information is generated by EndNote 20.

2. Name of data extractors and date of data extraction

Author(s)	Year	Title	Name of data extractors	date
E. Carnegie; D. Marchant; S. Towers; P. Ellison	2020	Beyond visual fixations and gaze behaviour. Using pupillometry to examine the mechanisms in the planning and motor performance of a golf putt	Scopus	2023/12/28
W. K. Lam; R. S. Masters; J. P. Maxwell	2010	Cognitive demands of error processing associated with preparation and execution of a motor skill	Scopus	2023/12/28
O. R. Runswick; M. Jewiss; B. Sharpe; J. S. North	2021	Context Affects Quiet Eye Duration and Motor Performance Independent of Cognitive Effort	Web of Science	2023/12/28
E. J. Stevenson; P. R. Hayes; S. J. Allison	2009	The effect of a carbohydrate-caffeine sports drink on simulated golf performance	EBSCOhost	2023/12/28
P. W. Mumford; A. C. Tribby; C. N. Poole; V. J. Dalbo; A. T. Scanlan; J. R. Moon; M. D. Roberts; K. C. Young	2016	Effect of Caffeine on Golf Performance and Fatigue during a Competitive Tournament	Pubmed	2023/12/28
E. Galanis; L. Nurkse; J. Kooijman; E. Papagiannis; A. Karathanasi; N. Comoutos; Y. Theodorakis; A. Hatzigeorgiadis	2022	Effects of a Strategic Self-Talk Intervention on Attention Functions and Performance in a Golf Task under Conditions of Ego Depletion	Web of Science	2023/12/28
Y. Nagashima; K. Ehara; Y. Ehara; A. Mitsume; K. Kubo; S. Mineo	2023	Effects of Continuous Carbohydrate Intake with Gummies during the Golf Round on Interstitial Glucose, Golf Performance, and Cognitive Performance of Competitive Golfers: A Randomized Repeated-Measures Crossover Design	Scopus	2023/12/28
M. Shin; Y. Kim; S. Park	2019	Effects of State Anxiety and Ego Depletion on Performance Change in Golf Putting: A Hierarchical Linear Model Application	EBSCOhost	2023/12/28
M. J. Campbell; A. P. Moran; N. Bargary; S. Surmon; L. Bressan; I. C. Kenny	2019	Pupillometry During Golf Putting: A New Window on the Cognitive Mechanisms Underlying Quiet Eye	Web of Science	2023/12/28
B. K. Doan; R. U. Newton; W. J. Kraemer; Y. H. Kwon; T. P. Scheet	2007	Salivary cortisol, testosterone, and T/C ratio responses during a 36-hole golf competition	Pubmed	2023/12/28

Literature information is generated by EndNote 20.

3. Confirmation that the study was eligible to be included in the review

As shown in **Table 2** of the manuscript, eligibility for inclusion followed the PICOS criteria (Population, Intervention, Comparison, Outcome, and Study design). A study was eligible for inclusion in the review if it met all of the following conditions simultaneously: (1) Participants had to be healthy individuals engaged in golf; (2) Any type of intervention was considered; (3) Comparison group includes any different intervention or no intervention; (4) Outcomes included levels of MF or any form of golf performance, encompassing physical, technical, cognitive, and tactical aspects; and (5) The study designs needed to include randomized controlled trials (RCT), non-randomized controlled trials (nRCT), and non-randomized non-controlled trials (nCT).

4. Handling of missing data

We addressed the issue of missing data based on the actual circumstances, with all authors jointly deciding on the approach for handling missing data. During the literature screening process, there were a few articles where the author and journal information was unclear, but other information was complete and did not affect our screening. In this systematic review, there was only one study for which the full text could not be obtained. After joint evaluation by all authors, it was determined that the missing data would not have a significant impact on the analysis results. Therefore, we chose to ignore this missing data.
